# Synthesis and Spectral Characterization of Benzo-[6,7][1,5]diazocino[2,1-*a*]isoindol-12-(14*H*)-one Derivatives

**DOI:** 10.3390/molecules21080967

**Published:** 2016-07-23

**Authors:** Jatinder P. Bassin, Bhavani Anagani, Christopher Benham, Madhu Goyal, Maryam Hashemian, Ute Gerhard

**Affiliations:** School of Life and Medical Sciences, University of Hertfordshire, Hatfield AL10 9AB, UK; bhavani.anagani@gmail.com (B.A.); c.d.benham@herts.ac.uk (C.B.); m.goyal@herts.ac.uk (M.G.); u.gerhard@herts.ac.uk (M.H.)

**Keywords:** diazocine, isoindole, isobenzofuran-1(3*H*)-ones, 2D-NMR

## Abstract

A simple synthetic route affording 27%–85% yields of benzo[6,7][1,5]diazocino[2,1-*a*]isoindol-12(14*H*)-one ring systems from readily available 3-(2-oxo-2-phenylethyl) isobenzofuran-1(3*H*)-ones and 2-(aminomethyl)aniline starting materials in toluene and catalysed by *p*-toluene-sulfonic acid is developed. The ^1^H- and ^13^C-NMR spectra of the final products were assigned using a variety of one and two-dimensional NMR experiments. The distinction between the two potential isomers of the final products was made on the basis of heteronuclear multiple bond connectivity (HMBC) NMR spectra.

## 1. Introduction

In recent years the synthesis and chemistry of medium ring heterocycles has attracted considerable attention because they are often present in biologically active natural products and because of their broad pharmacological profile [1]. Nitrogen-containing eight-membered heterocycles such as azocines and diazocines are known to exhibit a number of important biological properties [2,3,4,5]. The general strategies towards the synthesis of eight-membered heterocycles remain an active area of research and the most common synthetic approach to construct diazocine rings involves the conventional condensation reaction of 2-aminobenzophenones. However, this method is time-consuming, and the yield varies with different substrates. Furthermore, the syntheses of 2-aminobenzophenones can be fairly complicated and expensive [6]. As a result, synthetic strategies for diazocines preparation are limited [7].

**Figure 1:**
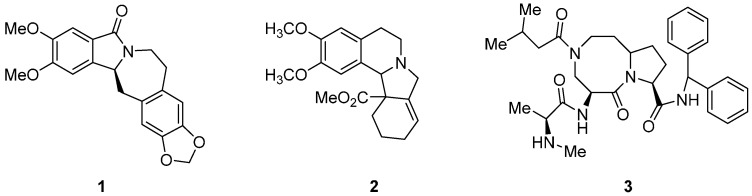
Biologically active isoindoles and fused diazocines **1**–**3**.

The isoindole ring system plays a key role in many pharmaceutical agents owing to their broad range of biological activities [8,9,10,11,12,13,14]. Isoindolinones are the core of many natural products and biologically active compounds such as the benzazepine alkaloids lennoxamine (**1**) and jamtine (**2**) (Figure 1). The literature shows that the occurrence of medium-sized rings with two nitrogen atoms in bio-active compounds increases the pharmaceutical strength and activities of the compounds. The [1,5]benzodiazocines are known as homologs of 1,4-benzodiazepines and inhibitors of 17β-hydroxysteroid dehydrogenase type 3. The 17β-hydroxysteroid dehydrogenases play key roles in the formation of active intracellular sex steroids [15]. Impairment of this testosterone-converting enzyme has been shown to be responsible for male pseudohermaphroditism [16], moreover, [1,5]benzodiazocine derivatives showed low to moderate ability to inhibit the 17β-hydroxysteroid dehydrogenase type 3 enzyme which can be used in the treatment of hormone-dependent cancer. Interest and research in the preparation of compounds containing eight-membered rings has increased considerably in recent years. However, the formation of these ring systems is a challenge for synthetic chemistry researchers. Due to unfavourable entropic and enthalpic effects, the ring closure to form eight-membered rings by intramolecular cyclisation reactions is often difficult in comparison to smaller sized rings. As a result, the usual synthetic strategies for the preparation of other ring systems cannot always be applied to eight-membered rings. Several conventional approaches such as intramolecular cyclization, intermolecular cyclization, palladium-catalyzed, Ugi Four-Center Three-Component coupling reaction (U-4C-3CR), use of microwave radiation, Morita–Baylis–Hillman reaction and intramolecular Friedel–Crafts strategies have been reported in the literature [17,18,19,20,21,22,23,24,25,26,27]. Despite the large number of literature reports on the conventional synthesis of diazocine skeletons, these conventional methods suffer from some drawbacks such as long reaction times, harsh reaction conditions, low-product yields, high cost, toxic by-products and use of toxic catalysts. Therefore, there is a need to introduce new and more efficient methods in order to develop the synthesis of medium-sized rings in the pharmaceutical industry. Interesting biological activities are shown by compounds containing five or six membered heterocyclic rings fused to diazocines [28,29,30,31,32,33,34]. Compound **3**, a potent and orally bioavailable Smac mimetic, inhibits cell growth and induces apoptosis in cancer cells and has been shown to be a potent antagonist of inhibitor of apoptosis proteins (IAPs). It is in phase 1 clinical trials for the treatment of human cancer [35,36,37].

## 2. Results and Discussion

Our interest in the synthesis of heterocyclic rings fused to medium sized rings led us to envisage a straightforward synthetic approach to the benzo[6,7][1,5]diazocine[2,1-*a*]isoindol-12-(14*H*)-one ring system [38,39]. A number of 3-(2-oxo-2-phenylethyl) isobenzofuran-1(3*H*)-ones **4a**–**m** were synthesized following the reported literature procedures [40,41,42,43,44,45,46,47,48,49,50,51] (Table 1). 

**Scheme 1:**
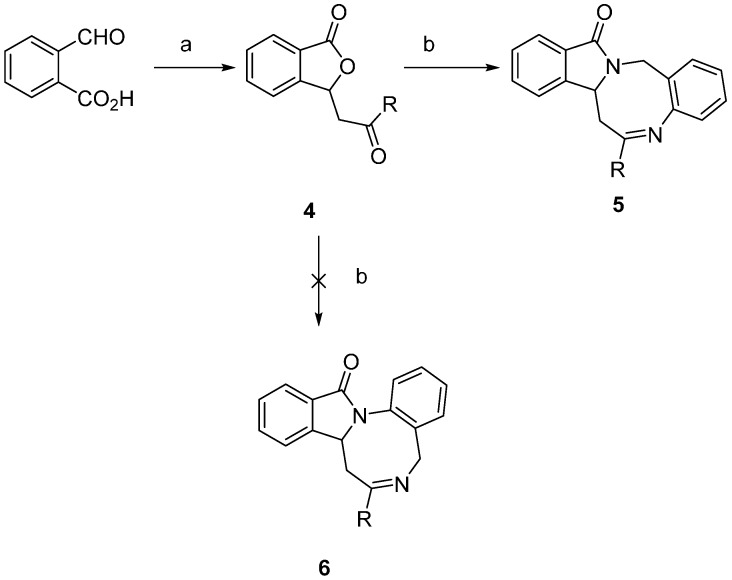
Synthesis of benzo[6,7][1,5]diazocine[2,1-*a*]isoindol-12(14H)-one **5a**–**5m**. *Reagents and conditions*: (a) RCOCH_3_, NaOH, RT. (b) 2-aminobenzylamine, *p*-TsOH, toluene, reflux 24 h.

All isobenzofuran-1(3*H*)-ones synthesized in this work, except compounds **4g** and **4k**, are known and their melting points and spectral characterization showed good agreement with the literature values. Compounds **4g** and **4k** were fully characterized by spectral data. Previous work had reported the reaction of hydrazine with a number of 3-(2-oxo-2-phenylethyl) isobenzofuran-1(3*H*)-ones to yield pyrazolo[5,1-*a*]isoindol-8-ones [52]. More recently the reaction of *o*-phenylenediamine with 3-(2-oxo-2-phenylethyl) isobenzofuran-1(3*H*)-ones has been reported to yield 7,7a-dihydro-12*H*-isoindolo[2,1-*a*][1,5]benzodiazepin-12-one derivatives [53]. We anticipated that the reaction of 3-(2-oxo-2-phenylethyl) isobenzofuran-1(3*H*)-ones **4** with 2-aminobenzylamine could yield either compound **5** or **6** (Scheme 1).

Benzo[6,7][1,5]diazocine[2,1-*a*]isoindol-12(14*H*)-one **5a**–**5m** were prepared in a simple two-step sequence. In the first step 3-(2-oxo-2-phenylethyl) isobenzofuran-1(3*H*)-ones **4a**–**4m** were prepared in moderate to good yields following a known reported literature method [47]. Reaction of **4a**–**4m** with 2-aminobenzylamine afforded **5a**–**5m** in moderate to good yields (27%–85%) (Table 2). We envisaged that either regioisomer **5** or **6** could form, depending upon the mechanistic pathway followed.

**Figure 2:**
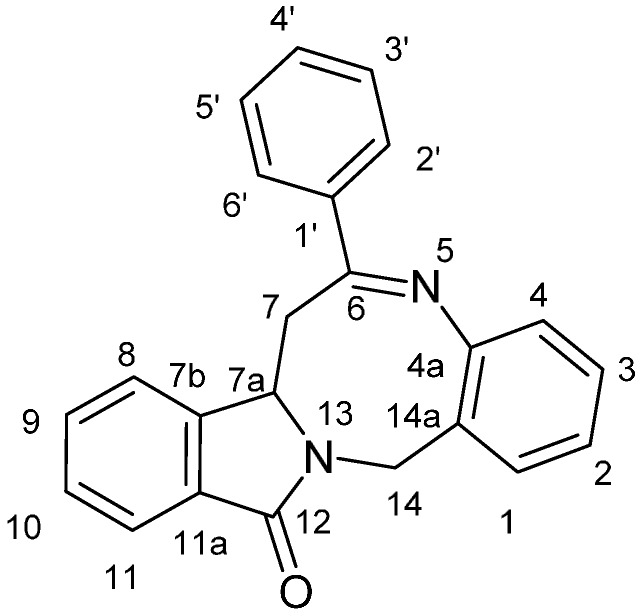
Numbering scheme of **5a** for the purpose of NMR assignments.

To investigate the regiochemistry of the reaction (Scheme 1), a full characterisation by NMR spectroscopy and assignment of compounds **5a**, **5b**, **5f** and **5h** were undertaken (Table 3 and Table 4). The numbering scheme is presented in Figure 2 and assignments of the proton and carbon spectra are summarized in Table 3 and Table 4, respectively.

**Figure 3:**
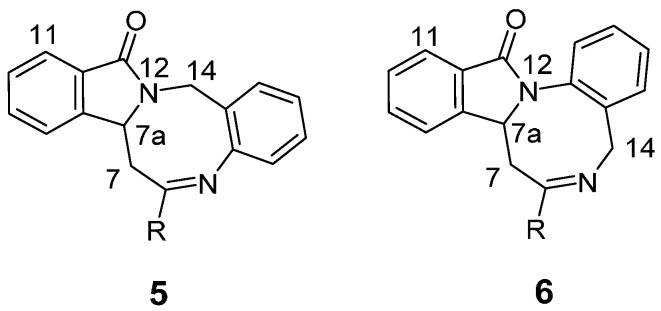
Potential reaction products formed.

The assignments were based on a combination of proton and carbon homo- and heteronuclear 2D NMR experiments. There is potential to form two isomers (compounds **5** and **6**, Scheme 1) in the final step of the reaction and the chemical shift assignments were made to establish the structure of the products formed. Many of the long range proton-carbon correlations observed in the heteronuclear multiple bond connectivity spectra (HMBC: see supplementary material) fit either of the two isomers (Figure 3). 

However, correlations from the methylene group protons H14 to the carbonyl group C12 are indicative of structure **5**. The assignment of C12 is confirmed by the long range proton-carbon correlation between H11 and C12.

Protons H7 and H14 from the methylene groups correlate with carbon 7a. Typically, correlations spanning two to three bonds are observed in the HMBC experiment and the methylene group protons next to the nitrogen in structure **6** are too far removed from carbon atoms C7a and C12 and, therefore, highly unlikely to be observed if structure **6** were formed in the reaction. The NMR analyses fully support the conclusion that isomer **5** was formed in this reaction. Other derivatives, where no 2D-NMR data were acquired follow the proton and carbon chemical shifts patterns of the fully characterized derivatives **5a**, **5b**, **5f** and **5h** and, therefore, the same regiochemistry was inferred.

## 3. Materials and Methods

### 3.1. General Information

All chemicals were purchased form Sigma Aldrich (Dorset, UK) or Merck (Nottingham, UK) and were used without further purification. Melting points were determined using a Gallenkamp melting point apparatus (Thermo Fisher Scientific, Paisley, UK) and are uncorrected. NMR spectra (at 600 MHz for protons and 151 MHz for ^13^C) were recorded on a ECA 600 MHz NMR instrument (JEOL Co Ltd., Tokyo, Japan) equipped with a 5 mm gradient broadband probe. Tetramethylsilane was used as internal standard and solvents as indicated. Chemical shifts were measured in ppm (δ) relative to TMS (0.00 ppm) or the residual solvent peaks. Coupling constants (J) are reported in Hertz (Hz). LC-MS spectra were obtained with a spectrometer equipped with an ESI source (Varian: 210 LC pumps × 2, 1200 L Quadrapole MS/MS, 410 autosampler) (Varian (now Agilent), Oxford, UK) using a gradient solvent system of A: Water/0.1% formic acid and B: acetonitrile/0.1% formic acid. Infrared spectra were recorded with a Varian 800 FT-IR spectrophotometer (Varian).

### 3.2. General Procedure for the Synthesis of 3-(2-Oxo-2-phenylethyl)isobenzofuran-1(3H)-ones ***4a**–**4m***

To a stirred solution of 2-carboxybenzaldehyde (15.0 g; 0.1 mol) dissolved in ethanol (50 mL) in a 1 L three-necked round bottom flask was added the relevant acetophenone (0.1 mol). The flask was immersed in a bath of crushed ice. Sodium hydroxide (75 mL, 1.75 M) was added dropwise and the mixture was stirred mechanically for 4 h. The resulting mixture was neutralized with dilute hydrochloric acid. Diethyl ether (approximately 50 mL) was added to precipitate the product. The crude product was filtered, washed with a small volume of distilled water and recrystallized from dichloromethane and ethanol.

*3-(2-Oxo-2-phenylethyl)isobenzofuran-1(3H)-one* (**4a**). White powder, yield 74%, m.p. 142–144 °C (lit. 146–147 °C [42]). IR (KBr) cm^−1^ 1685 (C=O), 1742 (C=O). ^1^H-NMR (CDCl_3_): δ (ppm) 7.91 (d, *J* = 7.6 Hz, 1H), 7.68 (t, *J* = 7.6 Hz, 1H), 7.49–7.62 (m, 4H), 7.44 (d, *J* = 1.4 Hz, 1H), 7.29–7.40 (m, 1H), 6.10 (t, *J* = 6.5 Hz, 1H), 3.64 (dd, *J* = 17.2, 6.9 Hz, 1H), 3.46 (dd, *J* = 17.9, 6.2 Hz, 1H). ^13^C-NMR (CDCl_3_) δ 195.74 (C=O), 169.87 (C=O), 149.47, 135.91, 134.04, 133.60, 129.17, 128.56, 127.91, 125.61, 125.42, 122.53, 76.80, 43.38. MS (ESI, *m*/*z*) 251.05 [M]^+^.

*3-(2-(4-Chlorophenyl)-2-oxoethyl)isobenzofuran-1(3H)-one* (**4b**). White powder, yield 65%, m.p. 139–141 °C (lit. 146 °C [48]). IR (KBr) cm^−1^ 1691 (C=O), 1742 (C=O). ^1^H-NMR (CDCl_3_): δ (ppm) 7.82–8.01 (m, 3H), 7.66 (t, *J* = 7.3 Hz, 1H), 7.54 (t, *J* = 6.9 Hz, 2H), 7.45 (d, *J* = 8.3 Hz, 2H), 6.15 (t, *J* = 6.9 Hz, 1H), 3.72 (dd, *J* = 17.4, 5.5 Hz, 1H), 3.36 (dd, *J* = 17.4, 6.4 Hz, 1H). ^13^C-NMR (CDCl_3_) δ 194.80 (C=O), 169.99 (C=O), 149.52, 140.46, 134.48, 134.31, 129.57, 129.53, 129.19, 125.89, 125.82, 122.70, 76.79, 43.66. MS (ESI *m*/*z*) 285.0 [M]^+^.

*3-(2-(3-Chlorophenyl)-2-oxoethyl)isobenzofuran-1(3H)-one* (**4c**). White powder, yield 76%, m.p. 126–127 °C (lit. 142–144 °C [51]). IR (KBr) cm^−1^ 1691 (C=O), 1747 (C=O). ^1^H-NMR (CDCl_3_): δ (ppm) 7.84–7.97 (m, 1H), 7.59–7.75 (m, 4H), 7.51–7.59 (m, 2H), 7.46 (td, *J* = 8.0, 5.0 Hz, 1H), 7.27–7.35 (m, 1H), 6.15 (t, *J* = 6.4 Hz, 1H), 3.73 (dd, *J* = 17.4, 6.0 Hz, 1H), 3.33–3.45 (m, 1H). ^13^C-NMR (CDCl_3_) δ 194.76 (C=O), 169.95 (C=O), 149.44, 137.63, 135.25, 134.31, 133.78, 130.18, 129.54, 128.23, 126.25, 125.83, 122.66, 76.79, 43.79. MS (ESI *m*/*z*) 285.0 [M]^+^.

*3-(2-(2-Chlorophenyl)-2-oxoethyl)isobenzofuran-1(3H)-one* (**4d**). White powder, yield 79%, m.p. 95–96 °C (lit. 91–92 °C [47]). IR (KBr) cm^−1^ 1678 (C=O), 1735 (C=O). ^1^H-NMR (CDCl_3_): δ (ppm) 7.85–7.95 (m, 1H), 7.62–7.74 (m, 1H), 7.48–7.62 (m, 3H), 7.38–7.48 (m, 2H), 7.29–7.38 (m, 1H), 6.07–6.17 (m, 1H), 3.59–3.77 (m, 1H), 3.44–3.58 (m, 1H). ^13^C-NMR (CDCl_3_) δ 198.52 (C=O), 169.95 (C=O), 149.27, 137.93, 134.30, 132.65, 131.31, 30.77, 129.64, 129.49, 127.21, 125.90, 125.82, 122.45, 76.79, 47.74. MS (ESI *m*/*z*) 286.9 [M]^+^.

*3-(2-(4-Bromophenyl)-2-oxoethyl)isobenzofuran-1(3H)-one* (**4e**). White powder, yield 45%, m.p. 144–146 °C (lit. 147–149 °C [42]). IR (KBr) cm^−1^ 1679 (C=O), 1740 (C=O). ^1^H-NMR (CDCl_3_): δ (ppm) 7.86–7.98 (m, 1H), 7.74–7.86 (m, 2H), 7.58–7.73 (m, 3H), 7.47–7.58 (m, 2H), 6.14 (t, *J* = 6.5 Hz, 1H), 3.71 (ddd, *J* = 17.7, 6.0, 1.4 Hz, 1H), 3.35 (ddd, *J* = 17.5, 7.0, 1.4 Hz, 1H). ^13^C-NMR (CDCl_3_) δ 195.00 (C=O), 169.98 (C=O), 149.51, 134.88, 134.31, 132.19, 129.64, 129.53, 129.22, 125.89, 125.83, 122.69, 76.79, 43.63. MS (ESI *m*/*z*) 331.1 [M]^+^.

*3-(2-(3-Bromophenyl)-2-oxoethyl)isobenzofuran-1(3H)-one* (**4f**). White crystals, yield 75%, m.p. 127–129 °C (lit. 124–127 °C [52]). IR (KBr) cm^−1^ 1688 (C=O), 1729 (C=O). ^1^H-NMR (CDCl_3_): δ (ppm) 8.08 (t, *J* = 1.8 Hz, 1H), 7.89–7.96 (m, 1H), 7.79–7.89 (m, 1H), 7.69–7.79 (m, 1H), 7.60–7.69 (m, 1H), 7.48–7.60 (m, 2H), 7.30–7.45 (m, 1H), 6.15 (t, *J* = 6.4 Hz, 1H), 3.68–3.80 (m, 1H), 3.37 (dd, *J* = 17.7, 7.1 Hz, 1H). ^13^C-NMR (CDCl_3_) δ 194.68 (C=O), 169.95 (C=O), 149.45, 137.82, 136.71, 134.32, 131.20, 130.42, 129.55, 126.70, 125.89, 125.84, 123.23, 122.67, 76.79, 43.77. MS (ESI *m*/*z*) 332.8 [M]^+^.

*3-(2-(2-Bromophenyl)-2-oxoethyl)isobenzofuran-1(3H)-one* (**4g**). White powder, yield 67%, m.p. 107–108 °C. IR (KBr) cm^−1^ 1684 (C=O), 1749 (C=O). ^1^H-NMR (CDCl_3_): δ (ppm) 7.78–7.97 (m, 1H), 7.63–7.78 (m, 1H), 7.49–7.63 (m, 3H), 7.42–7.49 (m, 1H), 7.34–7.42 (m, 1H), 7.31 (td, *J* = 7.8, 1.8 Hz, 1H), 6.03–6.17 (m, 1H), 3.61 (dd, *J* = 17.7, 6.6 Hz, 1H), 3.41–3.54 (m, 1H). ^13^C-NMR (CDCl_3_) δ 199.38 (C=O), 169.88 (C=O), 149.14, 140.13, 134.27, 133.90, 132.37, 129.47, 129.11, 127.65, 125.81, 125.73, 122.47, 118.87, 76.78, 47.25. MS (ESI *m*/*z*) 329.0 [M]^+^. Anal. Calcd. For: C_16_H_11_BrO_3_: C, 58.03; H, 3.35%. Found: C, 57.85; H, 3.59%.

*3-(2-(4-Fluorophenyl)-2-oxoethyl)isobenzofuran-1(3H)-one* (**4h**). White powder, yield 81%, m.p. 135–136 °C (lit. 130–133 °C [52]). IR (KBr) cm^−1^ 1680 (C=O), 1745 (C=O). ^1^H-NMR (CDCl_3_): δ (ppm) 7.94–8.06 (m, 2H), 7.91 (dd, *J* = 7.6, 1.1 Hz, 1H), 7.60–7.72 (m, 1H), 7.50–7.60 (m, 2H), 7.12–7.23 (m, 2H), 6.15 (t, *J* = 6.5 Hz, 1H), 3.73 (dd, *J* = 17.5, 5.8 Hz, 1H), 3.36 (dd, *J* = 17.7, 7.1 Hz, 1H). ^13^C-NMR (CDCl_3_) δ 194.40 (C=O), 170.03 (C=O), 167.01, 149.59, 134.30, 132.65, 130.93, 130.86, 129.50, 125.89, 125.80, 122.74, 116.10, 115.95, 76.79, 43.60. MS (ESI *m*/*z*) 268.3 [M]^+^.

*3-(2-(3-Fluorophenyl)-2-oxoethyl)isobenzofuran-1(3H)-one* (**4i**). White powder, yield 62%, m.p. 116–117 °C (lit. 114 °C [47]). IR (KBr) cm^−1^ 1684 (C=O), 1744 (C=O). ^1^H-NMR (CDCl_3_): δ (ppm) 7.86–8.02 (m, 2H), 7.61–7.74 (m, 1H), 7.49–7.61 (m, 3H), 7.20–7.34 (m, 1H), 7.10–7.20 (m, 1H), 6.12–6.22 (m, 1H), 3.66–3.81 (m, 1H), 3.45 (dd, *J* = 6.4, 3.2 Hz, 1H). ^13^C-NMR (CDCl_3_) δ 193.92 (C=O), 170.11 (C=O), 163.04, 161.34, 149.62, 135.50, 134.23, 130.68, 129.40, 126.00, 125.78, 124.74, 122.60, 116.20, 76.79, 48.44. MS (ESI *m*/*z*) 268.8 [M]^+^.

*3-(2-Oxo-2-(m-tolyl)ethyl)isobenzofuran-1(3H)-one* (**4j**). White powder, yield 56%, m.p. 108–109 °C (lit. 104–105 °C [47]). IR (KBr) cm^−1^ 1676 (C=O), 1768 (C=O). ^1^H-NMR (CDCl_3_): δ (ppm) 7.85–7.96 (m, 1H), 7.69–7.82 (m, 2H), 7.60–7.69 (m, 1H), 7.47–7.60 (m, 2H), 7.31–7.47 (m, 2H), 6.17 (dd, *J* = 7.6, 5.7 Hz, 1H), 3.76 (dd, *J* = 17.7, 5.7 Hz, 1H), 3.32–3.44 (m, 1H), 2.40 (s, 3H). ^13^C-NMR (CDCl_3_) δ 196.23 (C=O), 170.13(C=O), 149.81, 138.71, 136.22, 134.63, 134.24, 129.40, 128.69, 128.66, 125.74, 125.39, 122.83, 76.79, 43.76, 21.32. MS (ESI *m*/*z*): 265.1 [M]^+^.

*3-(2-Oxo-2-(o-tolyl)ethyl)isobenzofuran-1(3H)-one* (**4k**). White powder, yield 63%, m.p. 102–103 °C. IR (KBr) cm^−1^ 1688 (C=O), 1740 (C=O). ^1^H-NMR (CDCl_3_): δ (ppm) 7.88–7.99 (m, 1H), 7.75–7.88 (m, 2H), 7.60–7.74 (m, 1H), 7.48–7.60 (m, 2H), 7.21–7.33 (m, 2H), 6.10–6.22 (m, 1H), 3.75 (dd, *J* = 17.5, 5.6 Hz, 1H), 3.35 (dd, *J* = 17.5, 7.5 Hz, 1H), 2.41 (s, 3H). ^13^C-NMR (CDCl_3_) δ 199.24 (C=O), 170.10 (C=O), 149.71, 139.00, 136.45, 134.24, 132.33, 132.21, 129.41, 128.97, 125.96, 125.91, 125.80, 122.58, 76.79, 46.15, 21.63. MS (ESI *m*/*z*) 264.6 [M]^+^. Anal. Calcd. For: C_17_H_14_O_3_: C, 76.68; H, 5.30%. Found: C, 76.53; H, 5.22%.

*3-(2-(3-Methoxyphenyl)-2-oxethyl)isobenzofuran-1(3H)-one* (**4l**). White powder, yield 68%, m.p. 110–111 °C (lit. 134–135 °C [42]). IR (KBr) cm^−1^ 1676 (C=O), 1768 (C=O). ^1^H-NMR (CDCl_3_): δ (ppm) 7.91 (dd, *J* = 7.7, 1.0 Hz, 1H), 7.60–7.80 (m, 1H), 7.48–7.60 (m, 4H), 7.30–7.44 (m, 1H), 7.13 (dd, *J* = 8.3, 2.5, Hz, 1H), 6.14–6.22 (m, 1H), 3.85–4.02 (m, 3H), 3.73–3.79 (m, 1H), 3.35–3.44 (m, 1H). ^13^C-NMR (CDCl_3_) δ 196.23 (C=O), 170.13(C=O), 149.81, 138.71, 136.22, 134.63, 134.24, 129.40, 128.69, 128.66, 125.74, 125.39, 122.83, 76.79, 43.76, 21.32. MS (ESI *m*/*z*) 265.1 [M]^+^.

*3-(2-Oxo-2-(thiophen-2-yl)ethyl)isobenzofuran-1(3H)-one* (**4m**). White powder, yield 54%, m.p. 125–127 °C (lit. 125–127 °C) [49]. IR (KBr) cm^−1^ 1645 (C=O), 1767 (C=O). ^1^H-NMR (CDCl_3_): δ (ppm) 7.90 (d, *J* = 7.6 Hz, 1H), 7.73–7.84 (m, 1H), 7.58–7.73 (m, 2H), 7.48–7.58 (m, 1H), 7.32–7.48 (m, 1H), 7.19–7.32 (m, 1H), 7.02–7.19 (m, 1H), 6.11 (t, *J* = 6.5 Hz, 1H), 3.60–3.79 (m, 1H), 3.38–3.57 (m, 1H). ^13^C-NMR (CDCl_3_) δ 188.73 (C=O), 170.18 (C=O), 149.50, 143.44, 134.99, 134.46, 133.07, 129.61, 128.57, 125.84, 122.80, 44.18. MS (ESI *m*/*z*) 256.8 [M]^+^.

### 3.3. General Procedure for the Synthesis of Benzo[6,7][1,5]diazocino[2,1-a]isoindol-12(14H)-ones ***5a**–**5m***

3-(2-Oxo-2-phenylethyl)isobenzofuran-1(3*H*)-ones **4a**–**4m** (4.0 × 10^−3^ mol) were added to 2-aminobenzylamine (8.0 × 10^−3^ mol) dissolved in toluene (40 mL). To the mixture was added *p*-toluenesulfonic acid monohydrate (70 mg; 3.5 × 10^−4^ mol) as a catalyst. The mixture was refluxed with stirring for 24 h and the reaction monitored using TLC. The solution was left to cool to room temperature and the excess solvent was removed on a rotary evaporator. The resulting solid was filtered, dried and recrystallized from ethanol.

*(E)-6-Phenyl-7,7a-dihydrobenzo[6,7][1,5]diazocino[2,1-a]isoindol-12(14H)-one* (**5a**). Light yellow crystals, yield 74%, m.p. 203–204 °C. IR (KBr) cm^−1^ 1700 (C=O), 1623 (C=N). ^1^H-NMR (CDCl_3_): δ (ppm) 8.12–8.21 (m, 2H), 7.83 (d, *J* = 7.2 Hz, 1H), 7.76 (dd, *J* = 7.7, 1.5 Hz, 1H), 7.51–7.64 (m, 4H), 7.37–7.51 (m, 2H), 7.22–7.33 (m, 1H), 7.07–7.16 (m, 1H), 7.01 (dd, *J* = 7.7, 1.2 Hz, 1H), 5.25–5.34 (m, 1H), 4.53 (d, *J* = 10.7 Hz, 1H), 3.60–3.74 (m, 2H), 2.25–2.35 (m, 1H). ^13^C-NMR (CDCl_3_) δ 167.16, 165.68, 148.78, 143.90, 137.18, 132.62, 131.78, 131.64, 131.42, 129.13, 129.00, 128.84, 128.80, 127.80, 127.74, 126.16, 125.09, 123.99, 121.91, 121.14, 56.11, 42.02, 36.28. MS (ESI *m*/*z*) 339.3 [M]^+^. Anal. Calcd. For: C_23_H_18_N_2_O: C, 81.63; H, 5.36; N, 8.28%. Found: C, 81.55; H, 5.66; N, 8.21%.

*(E)-6-(4-Chlorophenyl)-7,7a-dihydrobenzo[6,7][1,5]diazocino[2,1-a]isoindol-12(14H)-one* (**5b**). Light yellow crystals, yield 75%, m.p. 198–199 °C. IR (KBr) cm^−1^ 1695 (C=O), 1653 (C=N). ^1^H-NMR (CDCl_3_): δ (ppm) 8.10 (d, *J* = 8.3 Hz, 2H), 7.78–7.88 (m, 1H), 7.65–7.78 (m, 1H), 7.50–7.64 (m, 4H), 7.46 (ddd, *J* = 7.8, 6.0, 2.3 Hz, 1H), 7.22–7.34 (m, 1H), 7.07–7.17 (m, 1H), 6.96–7.05 (m, 1H), 5.29 (d, *J* = 14.7 Hz, 1H), 4.50 (d, *J* = 10.5 Hz, 1H), 3.64 (d, *J* = 14.2 Hz, 1H), 3.57 (d, *J* = 13.3 Hz, 1H), 2.28 (dd, *J* = 13.8, 11.0 Hz, 1H). ^13^C-NMR (CDCl_3_) δ 167.13, 164.43, 148.50, 143.69, 137.76, 135.58, 132.59, 131.85, 131.68, 129.40, 129.34, 129.08, 129.04, 128.94, 128.84, 126.08, 125.28, 124.07, 121.84, 121.14, 56.30, 42.00, 36.19. MS (ESI *m*/*z*) 373.2 [M]^+^. Anal. Calcd. For: C_23_H_17_ClN_2_O: C, 74.09; H, 4.60; N, 7.51%. Found: C, 73.85; H, 4.50; N, 7.45%.

*(E)-6-(3-Chlorophenyl)-7,7a-dihydrobenzo[6,7][1,5]diazocino[2,1-a]isoindol-12(14H)-one* (**5c**). Light yellow crystals, 73%, m.p. 107–108 °C. IR (KBr) cm^−1^ 1699 (C=O), 1653 (C=N). ^1^H-NMR (CDCl_3_): δ (ppm) 8.18 (t, *J* = 2.1 Hz, 1H), 8.00 (dt, *J* = 7.8, 1.4 Hz, 1H), 7.81 (d, *J* = 7.6 Hz, 1H), 7.74 (dd, *J* = 7.7, 1.5 Hz, 1H), 7.43–7.62 (m, 5H), 7.22–7.35 (m, 1H), 7.13 (td, *J* = 7.6, 1.4 Hz, 1H), 7.00 (dd, *J* = 7.9, 1.4 Hz, 1H), 5.30 (d, *J* = 14.4 Hz, 1H), 4.51 (d, *J* = 10.7 Hz, 1H), 3.65 (d, *J* = 14.4 Hz, 1H), 3.57 (dd, *J* = 13.7, 1.4 Hz, 1H), 2.28 (dd, *J* = 13.6, 10.8 Hz, 1H). ^13^C-NMR (CDCl_3_) δ 167.03, 164.24, 148.26, 143.57, 138.90, 135.35, 132.47, 131.77, 131.59, 131.27, 130.20, 128.85, 128.77, 127.95, 125.93, 125.56, 125.29, 123.97, 121.80, 121.00, 56.13, 41.89, 36.23. MS (ESI *m*/*z*) 373.2 [M]^+^. Anal. Calcd. For: C_23_H_17_ClN_2_O: C, 74.09; H, 4.60; N, 7.51%. Found: C, 74.20; H, 4.55; N, 7.50%.

*(E)-6-(2-Chlorophenyl)-7,7a-dihydrobenzo[6,7][1,5]diazocino[2,1-a]isoindol-12(14H)-one* (**5d**). Light brown crystals, yield 73%, m.p. 191–192 °C. IR (KBr) cm^−1^ 1685 (C=O), 1552 (C=N). ^1^H-NMR (CDCl_3_,): δ (ppm) 7.74–7.87 (m, 1H), 7.31–7.50 (m, 5H), 7.18–7.31 (m, 2H), 6.99–7.18 (m, 2H), 6.56–6.74 (m, 2H), 4.98–5.15 (m, 2H), 4.37 (d, *J* = 15.1 Hz, 1H), 3.75 (dd, *J* = 17.5, 3.8 Hz, 1H), 3.23 (dd, *J* = 17.4, 8.1 Hz, 1H). ^13^C-NMR (CDCl_3_) δ 168.49, 167.14, 147.49, 143.58, 139.17, 132.35, 131.77, 131.60, 131.53, 130.83, 130.71, 130.19, 128.77, 128.65, 127.34, 126.00, 125.50, 123.76, 122.03, 121.20, 55.05, 41.81, 40.23. MS (ESI *m*/*z*) 373.2 [M]^+^. Anal. Calcd. For: C_23_H_17_ClN_2_O: C, 74.09; H, 4.60; N, 7.51%. Found: C, 74.00; H, 4.55; N, 7.50%.

*(E)-6-(4-Bromophenyl)-7,7a-dihydrobenzo[6,7][1,5]diazocino[2,1-a]isoindol-12(14H)-one* (**5e**). Light yellow crystals, yield 85%, m.p. 196–197 °C. IR (KBr) cm^−1^ 1691 (C=O), 1662 (C=N). ^1^H-NMR (CDCl_3_): δ (ppm) 7.68–7.88 (m, 2H), 7.51–7.68 (m, 2H), 7.33–7.51 (m, 4H), 7.20–7.33 (m, 2H), 7.14 (td, *J* = 7.6, 1.4 Hz, 1H), 7.03 (dd, *J* = 7.9, 1.4 Hz, 1H), 5.38 (d, *J* = 14.8 Hz, 1H), 4.46 (d, *J* = 10.7 Hz, 1H), 4.02 (d, *J* = 14.4 Hz, 1H), 3.51 (dd, *J* = 13.2, 1.5 Hz, 1H), 2.35 (dd, *J* = 13.2, 11.2 Hz, 1H). ^13^C-NMR (CDCl_3_) δ 167.05, 164.48, 148.39, 143.58, 135.93, 134.33, 132.47, 132.23, 132.04, 131.76, 131.58, 129.46, 129.14, 128.85, 128.76, 127.49, 125.20, 121.75, 121.56, 121.01, 56.19, 41.90, 36.05. MS (ESI *m*/*z*) 417.1[M]^+^. Anal. Calcd. For: C_23_H_17_BrN_2_O: C, 66.20; H, 4.11; N, 6.71%. Found: C, 66.22; H, 4.11; N, 6.70%.

*(E)-6-(3-Bromophenyl)-7,7a-dihydrobenzo[6,7][1,5]diazocino[2,1-a]isoindol-12(14H)-one* (**5f**). White crystals, yield 72%, m.p. 219–220 °C. IR (KBr) cm^−1^ 1698 (C=O), 1625 (C=N). ^1^H-NMR (CDCl_3_): δ (ppm) 8.34 (t, *J* = 1.8 Hz, 1H), 8.01–8.10 (m, 1H), 7.81 (d, *J* = 7.3 Hz, 1H), 7.66–7.78 (m, 2H), 7.52–7.65 (m, 2H), 7.37–7.52 (m, 2H), 7.22–7.35 (m, 1H), 7.10–7.18 (m, 1H), 6.98–7.09 (m, 1H), 5.29 (d, *J* = 14.7 Hz, 1H), 4.51 (d, *J* = 10.5 Hz, 1H), 3.65 (d, *J* = 14.7 Hz, 1H), 3.56 (d, *J* = 13.8 Hz, 1H), 2.28 (dd, *J* = 13.8, 11.0 Hz, 1H). ^13^C-NMR (CDCl_3_) δ 167.12, 164.23, 148.35, 143.66, 139.20, 134.29, 132.57, 131.88, 131.69, 131.02, 130.54, 128.96, 128.87, 126.09, 125.95, 125.40, 124.06, 123.57, 121.89, 121.11, 56.22, 41.99, 36.30. MS (ESI *m*/*z*) 417.1 [M]^+^. Anal. Calcd. For: C_23_H_17_BrN_2_O: C, 66.20; H, 4.11; N, 6.71%. Found: C, 66.15; H, 4.10; N, 6.65%.

*(E)-6-(2-Bromophenyl)-7,7a-dihydrobenz[6,7][1,5]diazocino[2,1-a]isoindol-12(14H)-one* (**5g**). Colourless needles, yield 27%, m.p. 137–138 °C. IR (KBr) cm^−1^ 1683 (C=O), 1653 (C=N). ^1^H-NMR (CDCl_3_): δ (ppm) 7.78–7.85 (m, 1H), 7.53–7.63 (m, 1H), 7.36–7.52 (m, 3H), 7.20–7.34 (m, 2H), 7.06–7.20 (m, 3H), 6.59–6.70 (m, 2H), 5.00–5.12 (m, 2H), 4.38 (d, *J* = 15.5 Hz, 1H), 3.74 (dd, *J* = 17.7, 3.6 Hz, 1H), 3.22 (dd, *J* = 17.5, 7.9 Hz, 1H). ^13^C-NMR (CDCl_3_) δ 168.75, 145.90, 145.47, 140.60, 133.83, 132.12, 131.92, 131.31 130.95, 129.54, 128.71, 128.50, 127.52, 123.79, 122.92, 119.63, 117.21, 115.75, 55.88, 44.71, 42.10. MS (ESI *m*/*z*) 419.1 [M]^+^. Anal. Calcd. For: C_23_H_17_BrN_2_O: C, 66.20; H, 4.11; N, 6.71%. Found: C, 66.15; H, 4.05; N, 6.58%.

*(E)-6-(4-Fluorophenyl)-7,7a-dihydrobenzo[6,7][1,5]diazocino[2,1-a]isoindol-12(14H)-one* (**5h**). Colourless needles, yield 72%, m.p. 136–137 °C. IR (KBr) cm^−1^ 1696 (C=O), 1662 (C=N). ^1^H-NMR (CDCl_3_): δ (ppm) 8.17 (dd, *J* = 8.9, 5.3 Hz, 2H), 7.81 (d, *J* = 7.8 Hz, 1H), 7.66–7.78 (m, 1H), 7.52–7.66 (m, 2H), 7.37–7.52 (m, 1H), 7.22–7.37 (m, 3H), 7.05–7.17 (m, 1H), 6.97–7.05 (m, 1H), 5.29 (d, *J* = 14.2 Hz, 1H), 4.51 (d, *J* = 10.5 Hz, 1H), 3.66 (d, *J* = 14.2 Hz, 1H), 3.58 (d, *J* = 13.8 Hz, 1H), 2.29 (dd, *J* = 13.5, 10.8 Hz, 1H). ^13^C-NMR (CDCl_3_) δ 167.13, 164.80, 164.35, 148.58, 143.75, 133.38, 132.60, 131.782, 131.66, 129.92, 129.86, 128.92, 128.83, 126.14, 125.19, 124.06, 121.84, 121.19, 116.23, 116.09, 56.33, 42.01, 36.24. MS (ESI *m*/*z*) 557.3 [M]^+^. Anal. Calcd. For: C_23_H_17_FN_2_O: C, 77.51; H, 4.81; N, 7.86%. Found: C, 77.34; H, 4.85; N, 7.76%.

*(E)-6-(3-Fluorophenyl)-7,7a-dihydrobenzo[6,7][1,5]diazocino[2,1-a]isoindol-12(14H)-one* (**5i**). Colourless crystals, yield 55%, m.p. 187–189 °C. IR (KBr) cm^−1^ 1701 (C=O), 1620 (C=N). ^1^H-NMR (CDCl_3_): δ (ppm) 8.12–8.20 (m, 2H), 7.81 (d, *J* = 7.2 Hz, 1H), 7.66–7.78 (m, 1H), 7.51–7.63 (m, 4H), 7.41–7.51 (m, 1H), 7.23–7.33 (m, 1H), 7.07–7.16 (m, 1H), 6.97–7.05 (m, 1H), 5.29 (d, *J* = 14.4 Hz, 1H), 4.53 (d, *J* = 11.0 Hz, 1H), 3.60–3.74 (m, 2H), 2.28 (dd, *J* = 13.6, 10.8 Hz, 1H). ^13^C-NMR (CDCl_3_) δ 168.78, 163.25, 167.15, 162.80, 147.68, 146.09, 145.87, 143.98, 131.87, 135.21, 131.69, 131.36, 130.90, 129.41, 128.35, 123.74, 122.91, 117.38, 115.69, 55.59, 45.80, 41.91. MS (ESI *m*/*z*) 357.3 [M]^+^. Anal. Calcd. For: C_23_H_17_FN_2_O: C, 77.51; H, 4.81; N, 7.86%. Found: C, 77.45; H, 4.75; N, 7.75%.

*(E)-6-(m-Tolyl)-7,7a-dihydrobenzo[6,7][1,5]diazocino[2,1-a]isoindol-12(14H)-one* (**5j**). Colourless crystals, yield 68%, m.p. 150–151 °C. IR (KBr) cm^−1^ 1706 (C=O), 1680 (C=N); ^1^H-NMR (CDCl_3_): δ (ppm) 7.89 (d, *J* = 7.6 Hz, 2H), 7.52–7.70 (m, 3H), 7.09–7.21 (m, 2H), 6.93 (td, *J* = 7.5, 1.2 Hz, 1H), 6.80 (d, *J* = 7.9 Hz, 2H), 5.51 (d, *J* = 4.1 Hz, 1H), 5.21 (d, *J* = 17.2 Hz, 1H), 4.57 (d, *J* = 16.8 Hz, 1H), 4.12–4.30 (m, 1H), 3.48 (s, 2H), 0.95–1.13 (m, 1H). ^13^C-NMR (CDCl_3_) δ 166.38, 149.79, 148.69, 143.84, 132.14, 131.85, 131.51, 128.83, 128.70, 128.42, 126.07, 125.73, 125.39, 124.96, 124.64, 124.13, 123.88, 122.81, 121.14, 55.59, 45.80, 41.91, 21.31. MS (ESI *m*/*z*) 353.3 [M]^+^. Anal. Calcd. For: C_24_H_20_N_2_O: C, 81.79; H, 5.72; N, 7.95%. Found: C, 81.54; H, 5.53; N, 7.68%.

*(E)-6-(o-Tolyl)-7,7a-dihydrobenzo[6,7][1,5]diazocino[2,1-a]isoindol-12(14H)-one* (**5k**). Light yellow crystals, yield 40%, m.p. 155–156 °C. IR (KBr) cm^−1^ 1687 (C=O), 1298 (C-O), 1623 (C=N). ^1^H-NMR (CDCl_3_): δ (ppm) 8.05 (d, *J* = 8.2 Hz, 2H), 7.77–7.88 (m, 2H), 7.73 (dd, *J* = 7.6, 1.7 Hz, 1H), 7.50–7.61 (m, 1H), 7.40–7.50 (m, 2H), 7.36 (d, *J* = 8.2 Hz, 1H), 7.21–7.32 (m, 1H), 7.10 (td, *J* = 7.6, 1.4 Hz, 1H), 7.00 (dd, *J* = 7.7, 1.2 Hz, 1H), 5.27 (d, *J* = 14.4 Hz, 1H), 4.52 (d, *J* = 10.7 Hz, 1H), 3.68 (d, *J* = 14.4 Hz, 1H), 3.61 (dd, *J* = 13.7, 1.4 Hz, 1H), 2.43–2.54 (m, 3H), 2.26 (dd, *J* = 13.6, 10.8 Hz, 1H). ^13^C-NMR (CDCl_3_) δ 167.08, 165.44, 148.78, 143.86, 141.81, 134.22, 131.66, 131.50, 129.72, 129.38, 128.67, 128.27, 127.63, 126.14, 125.71, 124.85, 123.87, 122.84, 121.80, 121.17, 56.44, 41.92, 36.06, 21.43. MS (ESI *m*/*z*) 353.3. Anal. Calcd. For: C_24_H_20_N_2_O: C, 81.79; H, 5.72; N, 7.95%. Found: C, 81.50; H, 5.58; N, 7.90%.

*(E)-6-(3-Methoxyphenyl)-7,7a-dihydrobenzo[6,7][1,5]diazocino[2,1-a]isoindol-12(14H)-one* (**5l**). Light yellow crystals, yield 62%, m.p. 210–211 °C. IR (NKBr) cm^−1^ 1692 (C=O), 1265 (C-O), 1616 (C=N). ^1^H-NMR (CDCl_3_): δ (ppm) 7.80 (d, *J* = 7.6 Hz, 1H), 7.71–7.78 (m, 2H), 7.63–7.71 (m, 1H), 7.52–7.63 (m, 2H), 7.38–7.52 (m, 3H), 7.22–7.34 (m, 1H), 7.06–7.17 (m, 1H), 7.01 (dd, *J* = 7.9, 1.4 Hz, 1H), 5.29 (d, *J* = 14.4 Hz, 1H), 4.54 (d, *J* = 10.7 Hz, 1H), 3.92 (s, 3H), 3.69 (d, *J* = 14.4 Hz, 1H), 3.61 (dd, *J* = 13.7, 1.4 Hz, 1H), 2.27 (dd, *J* = 13.6, 10.8 Hz, 1H). ^13^C-NMR (CDCl_3_) δ 167.06, 165.36, 160.20, 148.62, 143.81, 138.49, 132.51, 131.70, 131.55, 129.91, 128.73, 128.70, 126.08, 125.01, 123.88, 121.82, 121.08, 119.90, 117.40, 112.80, 56.40, 55.50, 41.92, 36.29. MS (ESI *m*/*z*) 369.3 [M]^+^. Anal. Calcd. For: C_24_H_20_N_2_O_2_: C, 78.24; H, 5.47; N, 7.60%. Found: C, 78.15; H, 5.45; N, 7.55%.

*(E)-6-(Thiophen-2-yl)-7,7a-dihydrobenzo[6,7][1,5]diazocino[2,1-a]isoindol-12(14H)-one* (**5m**). Light yellow crystals, yield 32%, m.p. 279–280 °C. IR (KBr) cm^−1^ 1694 (C=O), 1661 (C=N). ^1^H-NMR (CDCl_3_): δ (ppm) 7.78–7.86 (m, 1H), 7.33–7.52 (m, 3H), 7.15–7.30 (m, 2H), 7.04–7.15 (m, 2H), 6.56–6.69 (m, 2H), 4.95–5.13 (m, 2H), 4.36–4.54 (m, 1H), 3.67–3.78 (m, 1H), 3.21 (dd, *J* = 17.4, 8.0 Hz, 1H). ^13^C-NMR (CDCl_3_) δ 167.10, 160.69, 144.47, 143.90, 143.73, 132.62, 131.82, 131.66, 131.50, 128.90, 128.77, 128.50, 128.09, 126.80, 125.34, 124.04, 121.90, 121.61, 56.95, 42.08, 37.31. MS (ESI *m*/*z*) 345.2 [M]^+^. Anal. Calcd. For: C_21_H_16_N_2_OS: C, 73.23; H, 4.68; N, 8.13%. Found: C, 73.05; H, 4.55; N, 8.05%.

## 4. Conclusions

In conclusion, we have developed a simple two-step synthetic route to the benzo[6,7][1,5]diazocino [2,1-*a*]isoindole fused ring system from readily available starting materials. A number of 3-(2-oxo-2-phenylethyl)isobenzofuran-1(3*H*)-ones **4a**–**4m** were reacted in boiling toluene with 2-aminobenzylamine under *para*-toluenesulfonic acid catalysis to yield the fused diazocine derivatives **5a**–**5m** in 27%–85% yields. The structure of the regioisomer formed has been confirmed unambiguously by rigorous multinuclear HMBC measurements. Biological evaluation of the synthesized compounds are currently under investigation and will be reported in future communications.

## Figures and Tables

**Table 1:** Formation of isobenzofuran-1(3*H*)-ones **4a**–**4m**.

Compound	R	Yield (%)	Melting Points (°C)	
			Found	Literature [Reference]
**4a**	C_6_H_5_	74	142–44	146–147 [42]
**4b**	4-ClC_6_H_4_	65	139–41	146 [48]
**4c**	3-ClC_6_H_4_	76	136–37	142–144 [51]
**4d**	2-ClC_6_H_4_	79	95–96	91–92 [47]
**4e**	4-BrC_6_H_4_	45	144–45	147–149 [42]
**4f**	3-BrC_6_H_4_	75	127–29	124–127 [52]
**4g**	2-BrC_6_H_4_	67	107–108	—
**4h**	4-FC_6_H_4_	81	135–36	130–133 [52]
**4i**	2-FC_6_H_4_	62	116–117	114 [47]
**4j**	3-CH_3_-C_6_H_4_	56	108–109	104–105 [47]
**4k**	2-CH_3_C_6_H_4_	63	102–103	—
**4l**	3-CH_3_OC_6_H_4_	68	110–111	134–135 [42]
**4m**	2-Thienyl	54	135–137	138 [49]

**Table 2:** Formation of benzo[6,7][1,5]diazocine[2,1-*a*]isoindol-12(14*H*)-ones **5a**–**5m**.

R	Compound	Yield (%)
C_6_H_5_	**5a**	74
4-ClC_6_H_4_	**5b**	75
3-ClC_6_H_4_	**5c**	73
2-ClC_6_H_4_	**5d**	73
4-BrC_6_H_4_	**5e**	85
3-BrC_6_H_4_	**5f**	72
2-BrC_6_H_4_	**5g**	27
4-FC_6_H_4_	**5h**	72
3-FC_6_H_4_	**5i**	55
3-CH_3_C_6_H_4_	**5j**	68
2-CH_3_C_6_H_4_	**5k**	40
3-CH_3_OC_6_H_4_	**5l**	62
2-Thienyl	**5m**	32

**Table 3:** Proton NMR assignments of compounds **5a**, **5b**, **5f** and **5h** (chemical shift, ppm) **^[a]^**.

Proton ^[b]^	Compound 5a	Compound 5b	Compound 5f	Compound 5h
7<‘>	2.30	2.30	2.30	2.31
7<‘‘>	3.66	3.59	3.58	3.60
14<‘>	3.71	3.66	3.67	3.68
7a	4.55	4.52	4.53	4.53
14<‘‘>	5.31	5.31	5.32	5.31
4	7.03	7.02	7.02	7.02
2	7.13	7.14	7.15	7.14
3	7.30	7.30	7.31	7.23–7.33
10	7.47	7.49	7.43–7.51	7.48
9	7.52–7.65	7.53–7.63	7.55–7.63	7.55–7.62
8	7.52–7.65	7.53–7.63	7.55–7.63	7.55–7.62
4’	7.52–7.65	—	7.72	—
3’	7.52–7.65	7.53–7.63	—	7.23–7.33
5’	7.52–7.65	7.53–7.63	7.43–7.51	7.23–7.33
1	7.76	7.76	7.76	7.76
11	7.83	7.83	7.83	7.83
2’	8.18	8.13	8.36	8.19
6’	8.18	8.13	8.07	8.19

^[a]^ All chemical shifts quoted were referenced to the residual solvent signal of CDCl_3_; ^[b]^ <‘> and <‘‘> represent germinal protons with different chemical shifts.

**Table 4:** Carbon NMR assignments of compounds **5a**, **5b**, **5f** and **5h** (chemical shifts, ppm) **^[a]^**.

Carbon	Compound 5a	Compound 5b	Compound 5f	Compound 5h	
7	36.28	36.19	36.30	36.24	
14	42.02	42.00	41.99	42.01	
7a	56.11	56.30	56.22	56.33	
4	121.21	121.14	121.11	121.19	
8	121.91	121.84	121.89	121.84	
11	123.99	124.07	124.06	124.06	
2	125.09	125.28	125.40	125.19	
14a	126.16	126.08	125.95	126.14	
2’	127.74	129.04	131.02	129.92	^3^*J*_C-F_ 8.7 Hz
6’	127.80	129.08	126.09	129.86	^3^*J*_C-F_ 8.7 Hz
3	128.80	128.84	128.87	128.83	
10	128.84	128.94	128.96	128.92	
3’	129.00	129.40	123.57	116.09	^2^*J*_C-F_ 21.7 Hz
5’	129.13	129.34	130.54	116.23	^2^*J*_C-F_ 21.7 Hz
4’	131.42	137.76	134.29	164.80	^1^*J*_C-F_ 252.9 Hz
1	131.64	131.68	131.69	131.66	
9	131.78	131.85	131.88	131.82	
11a	132.62	132.59	132.57	132.60	
1’	137.18	135.58	139.20	133.38	
7b	143.90	143.69	143.66	143.75	
4a	148.78	148.50	148.35	148.58	
6	165.68	164.43	164.23	164.35	
12	167.16	167.12	167.12	167.13	

^[a]^ All chemical shifts quoted were referenced to the carbon signal of CDCl_3_.

## Supplementary Materials

The following are available online at www.mdpi.com/1420-3049/21/8/967/s1.
