# Supplementary material for: Synthesis and Spectral Characterization of Benzo-[6,7][1,5]diazocino[2,1-a]isoindol-12-(14H)-one Derivatives
Source: Molecules. 2016 Jul 23;21(8):967. doi: 10.3390/molecules21080967 (PMC6274267; doi:10.3390/molecules21080967)
Supplement: Supplementary file 1 [file molecules-21-00967-s001.pdf]

Molecules **2016**, *21*, 967; doi:molecules21080967

Supplementary Materials: Synthesis and Spectral Characterization of Benzo[6,7][1,5]diazocino[2,1-a]isoindol-12-(14H)-one Derivatives

Jatinder P. Bassin, Bhavani Anagani, Christopher Benham, Madhu Goyal, Maryam Hashemian and Ute Gerhard

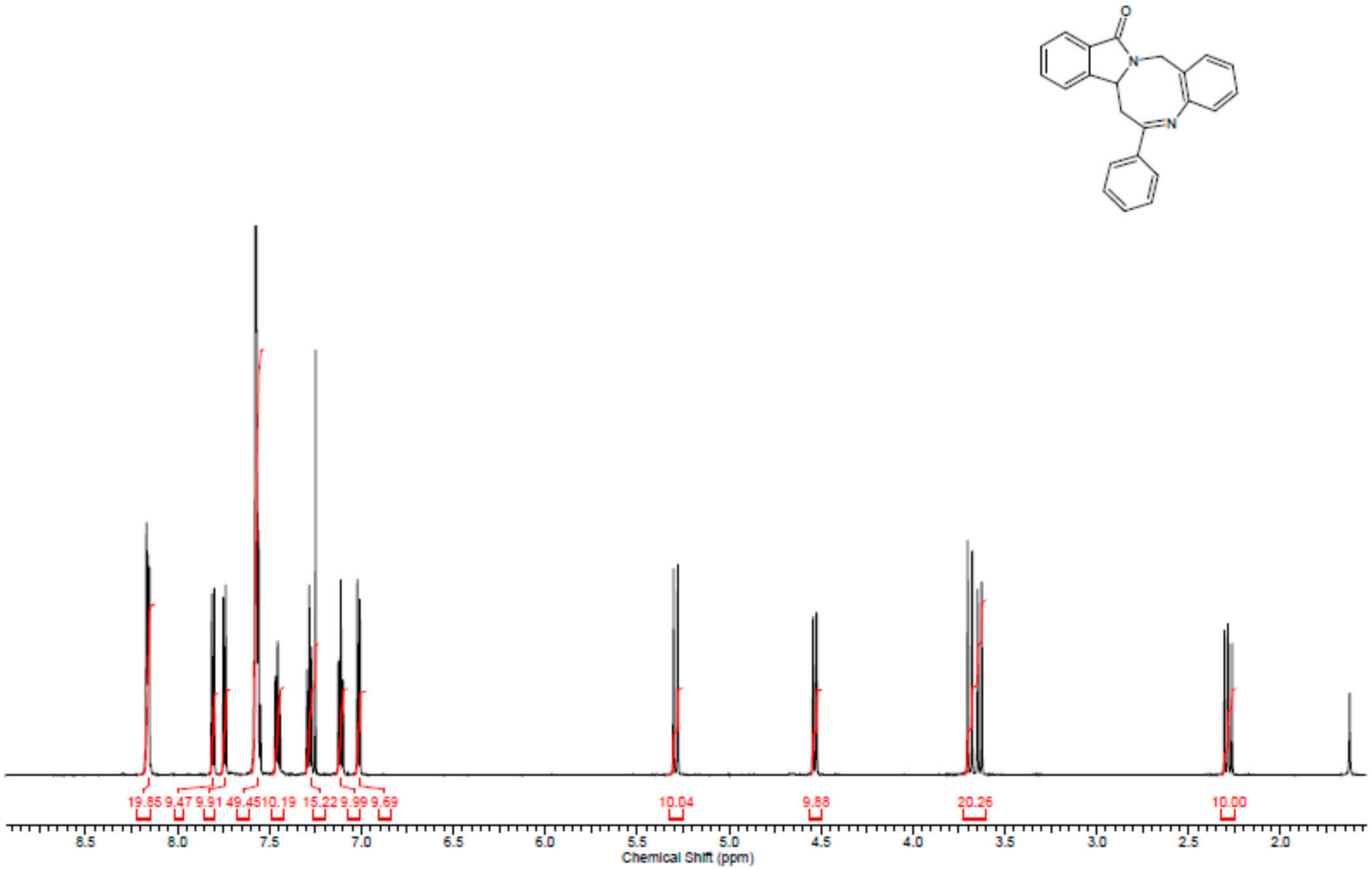

Figure S1. ^{1}H-NMR for compound **5a**.

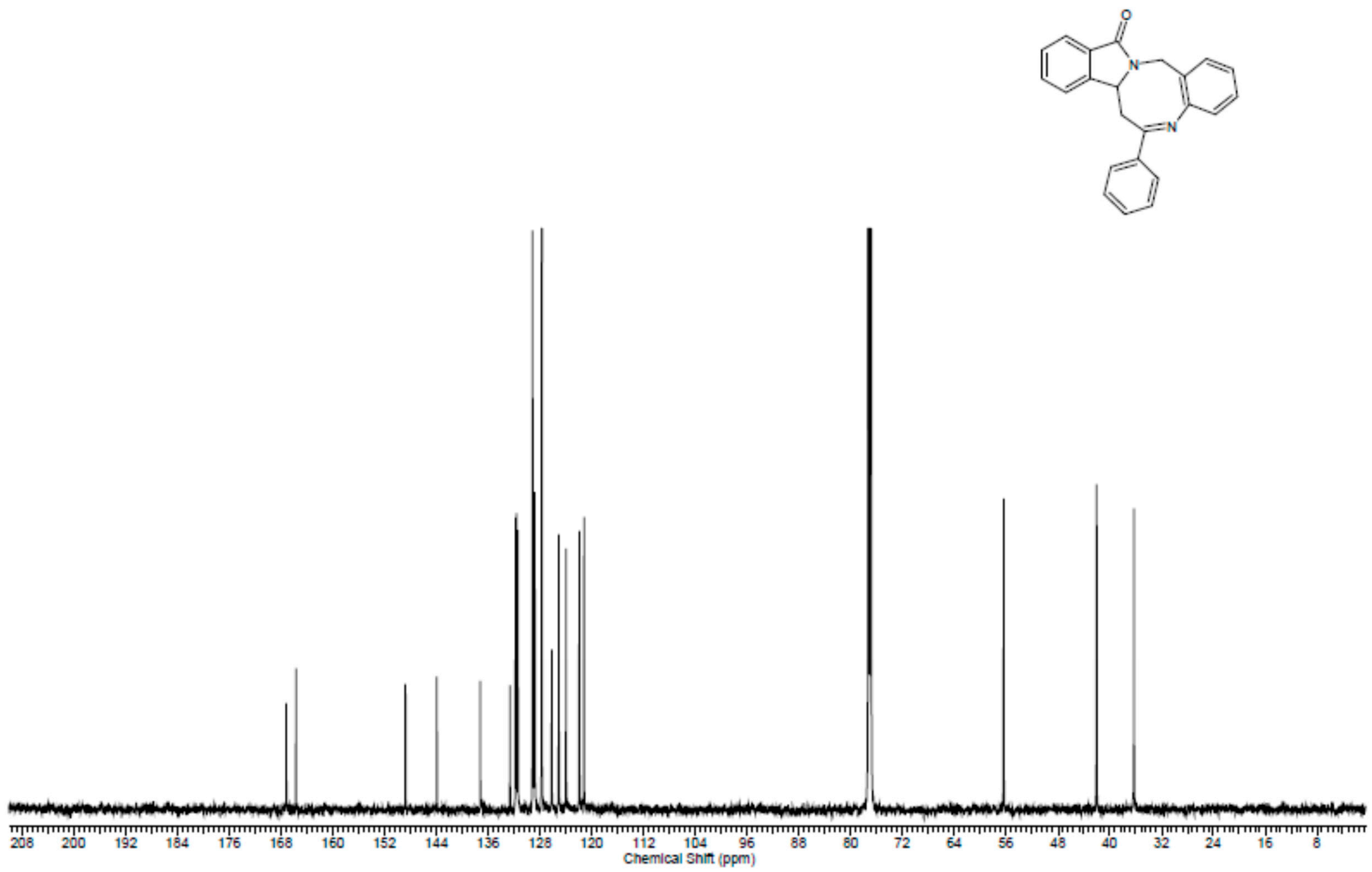

Figure S2. ^{13}C-NMR for compound **5a**.

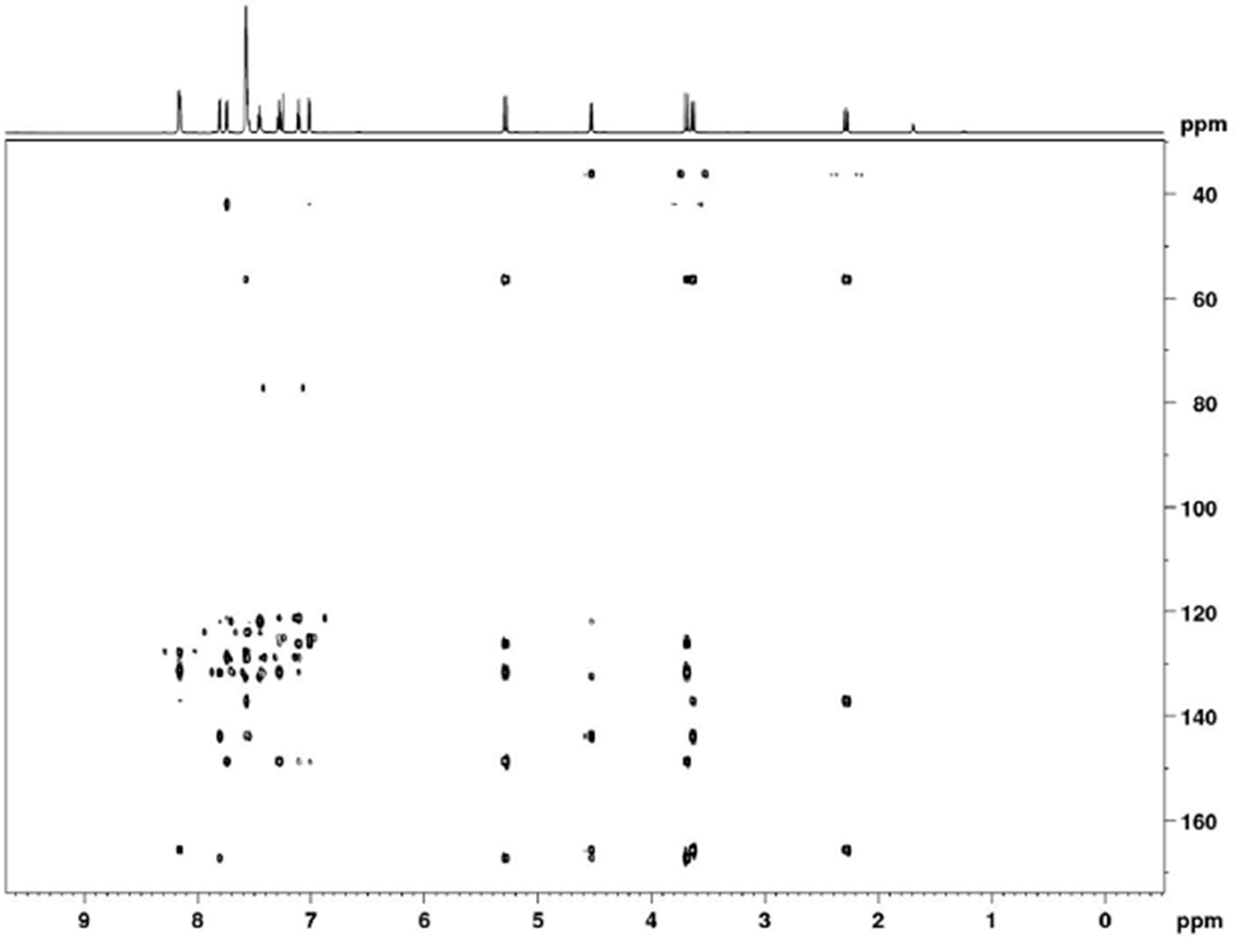

Figure S3. HMBC for compound **5a**.

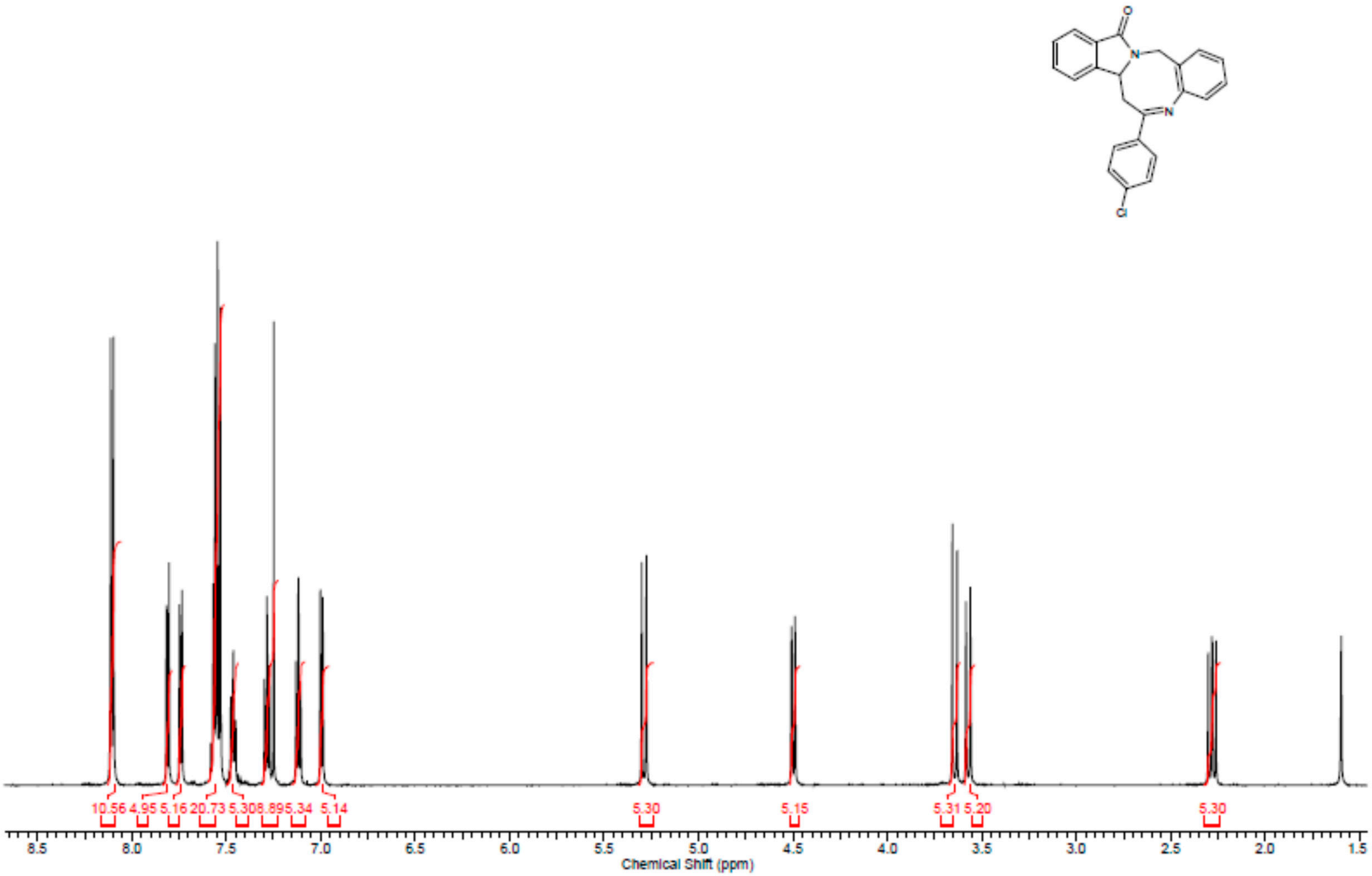

Figure S4. ^{1}H-NMR for compound **5b**.

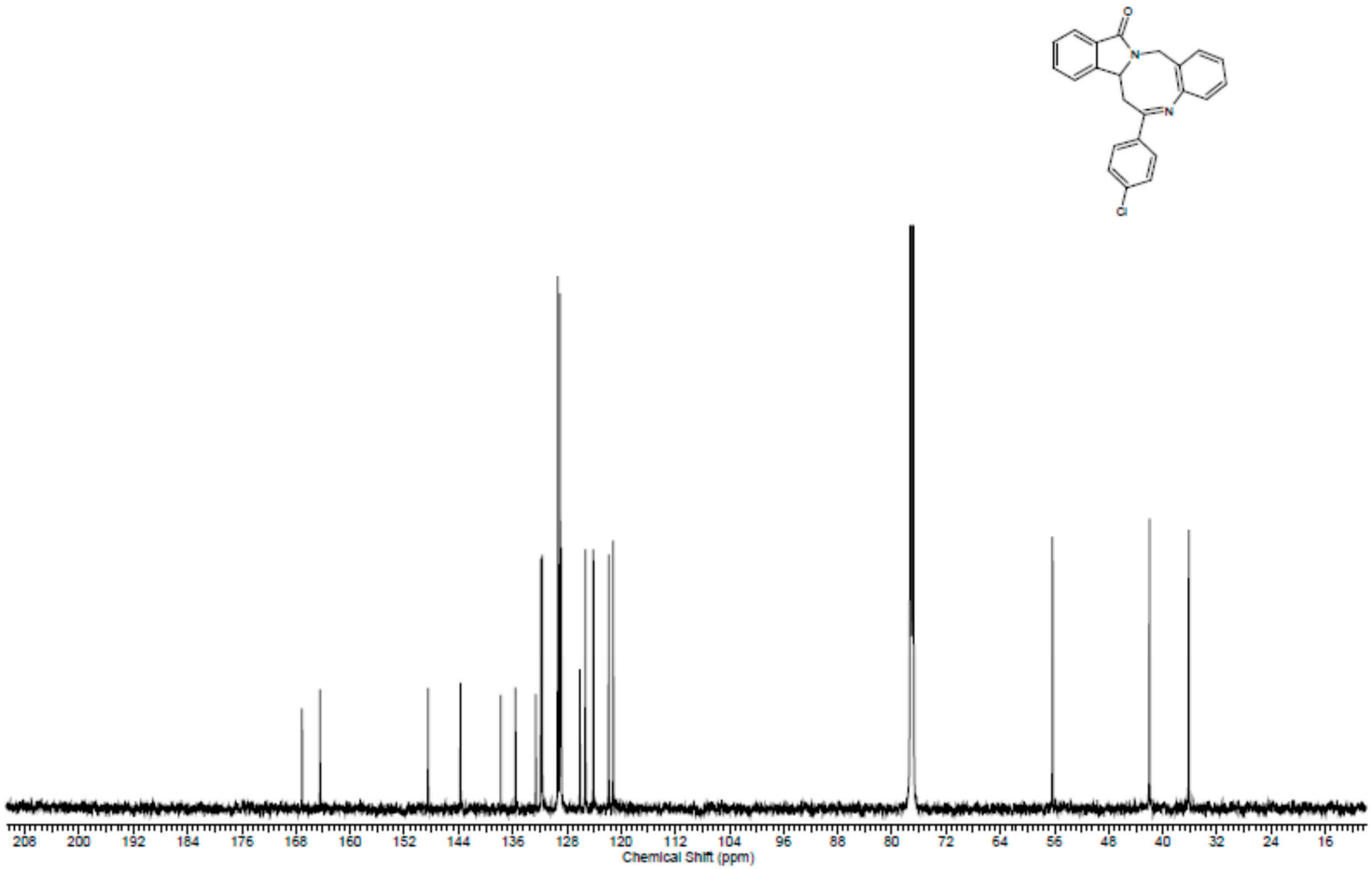

Figure S5. ^{13}C-NMRfor compound **5b**.

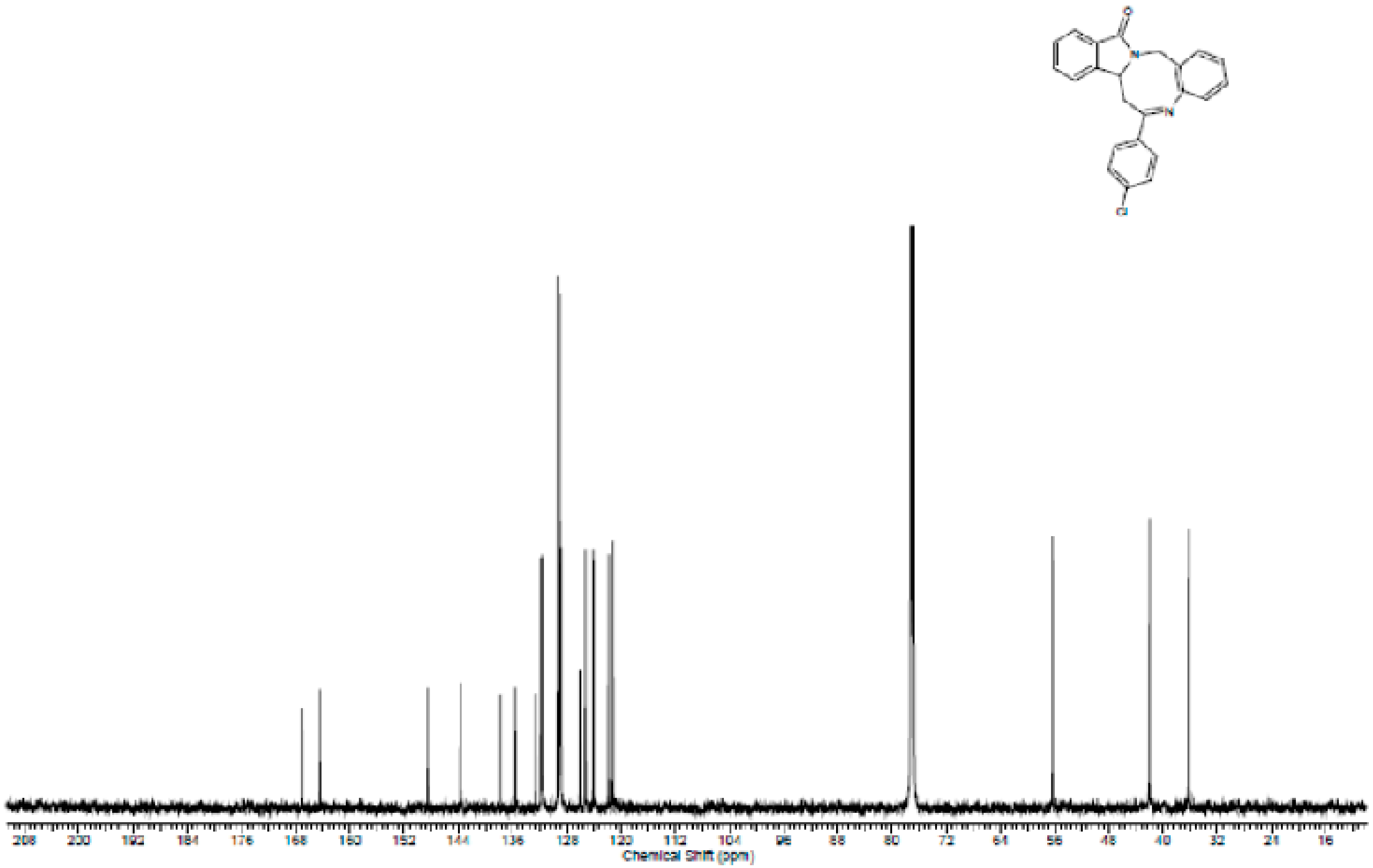

Figure S6. HMBC for compound **5b**.

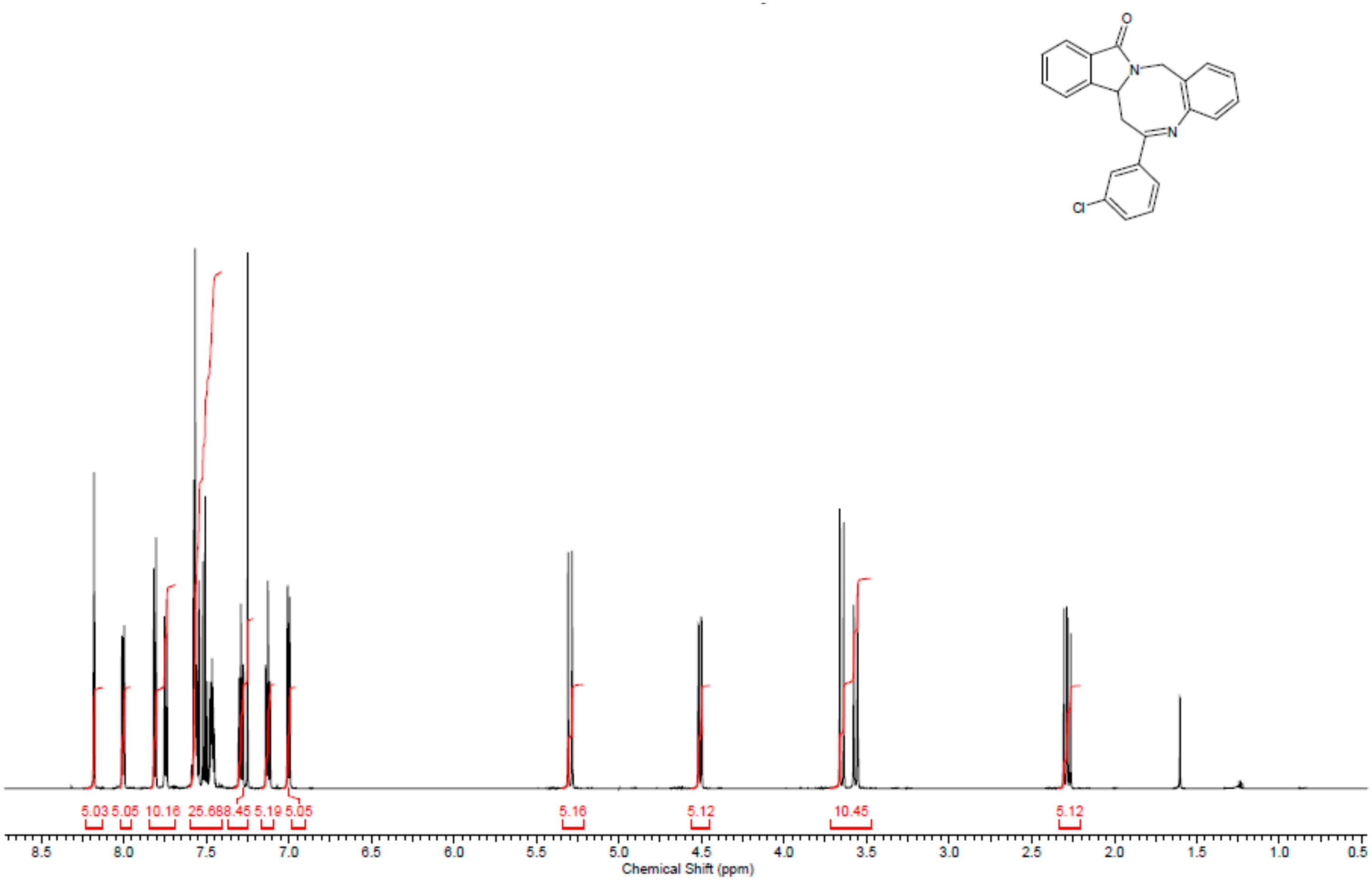

Figure S7. ^{1}H-NMR for compound **5c**.

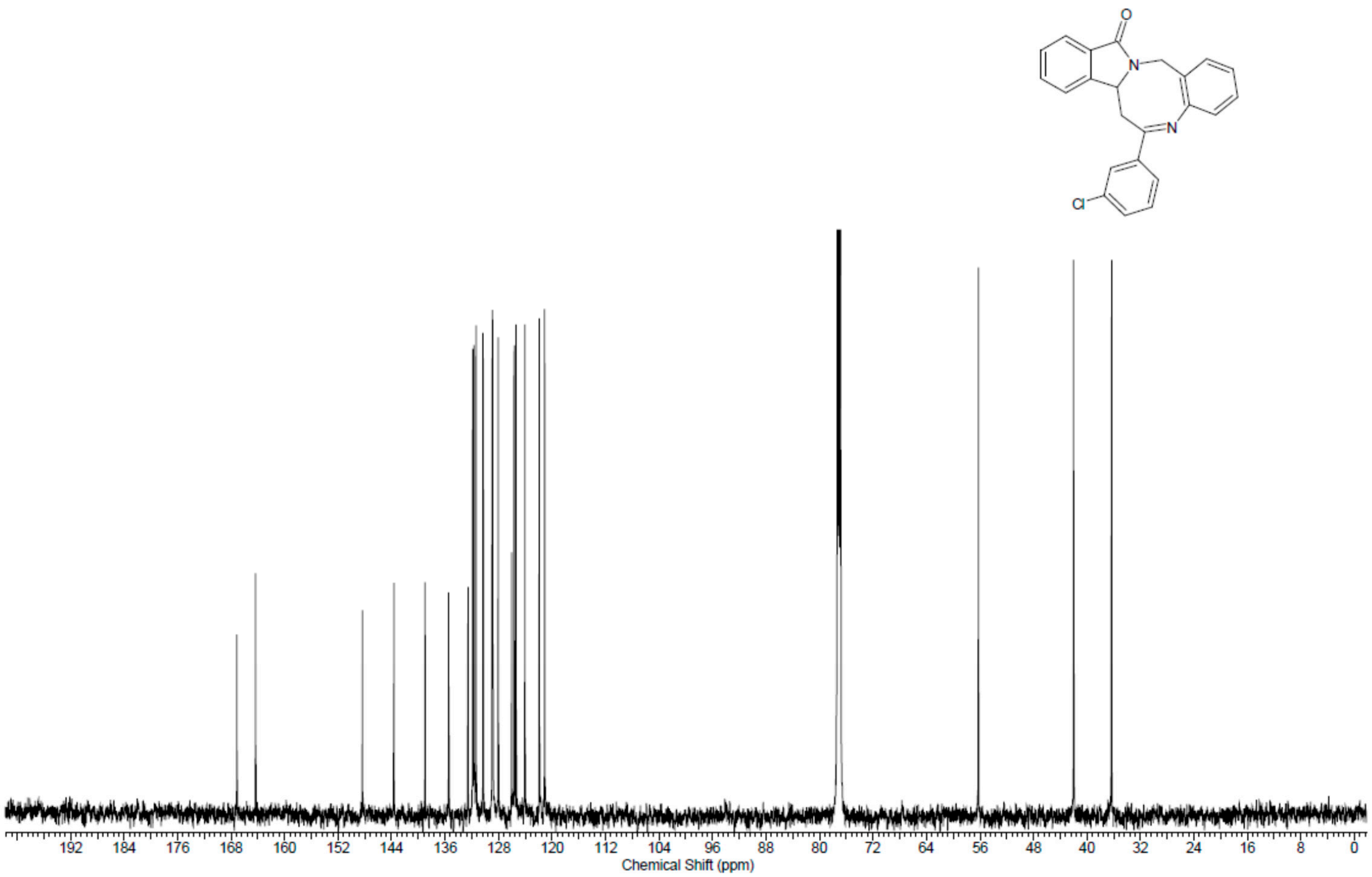

Figure S8. ^{13}C-NMRfor compound **5c**.

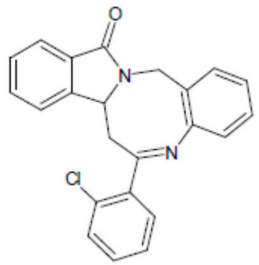

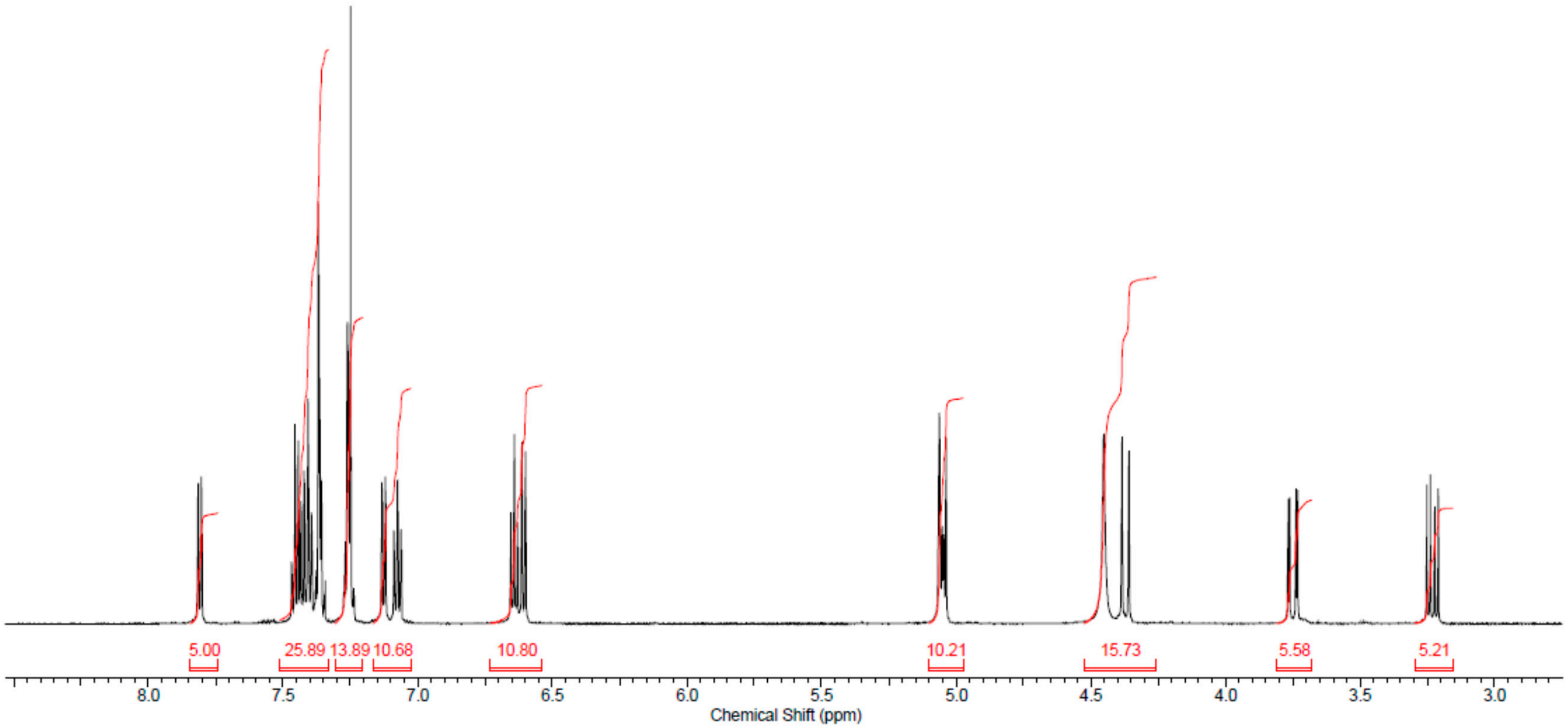

Figure S9. ^{1}H-NMR for compound **5d**.

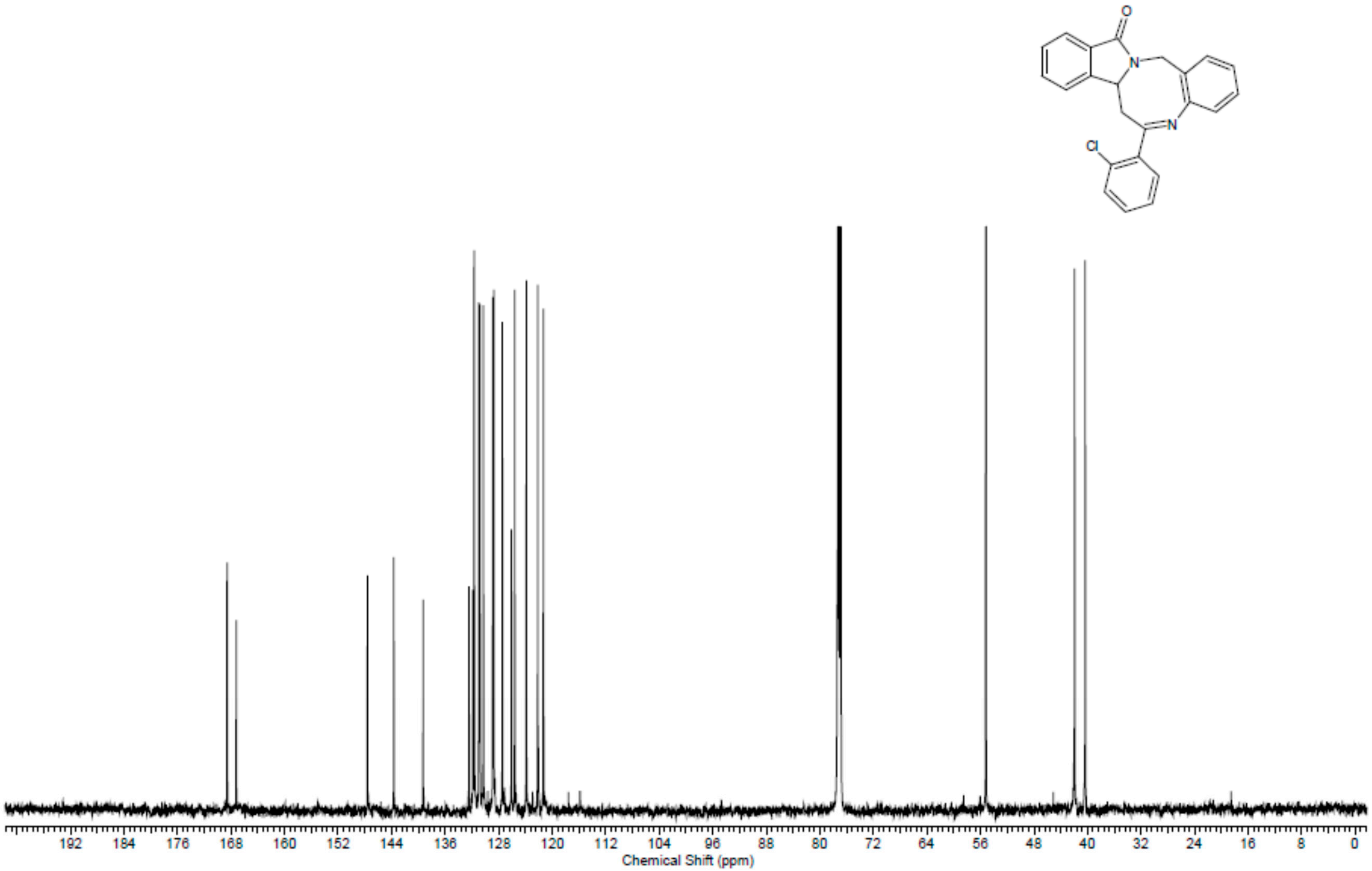

Figure S10. ^{13}C-NMRfor compound **5d**.

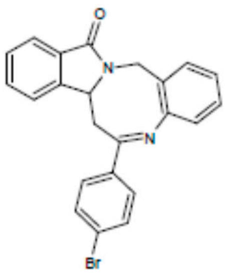

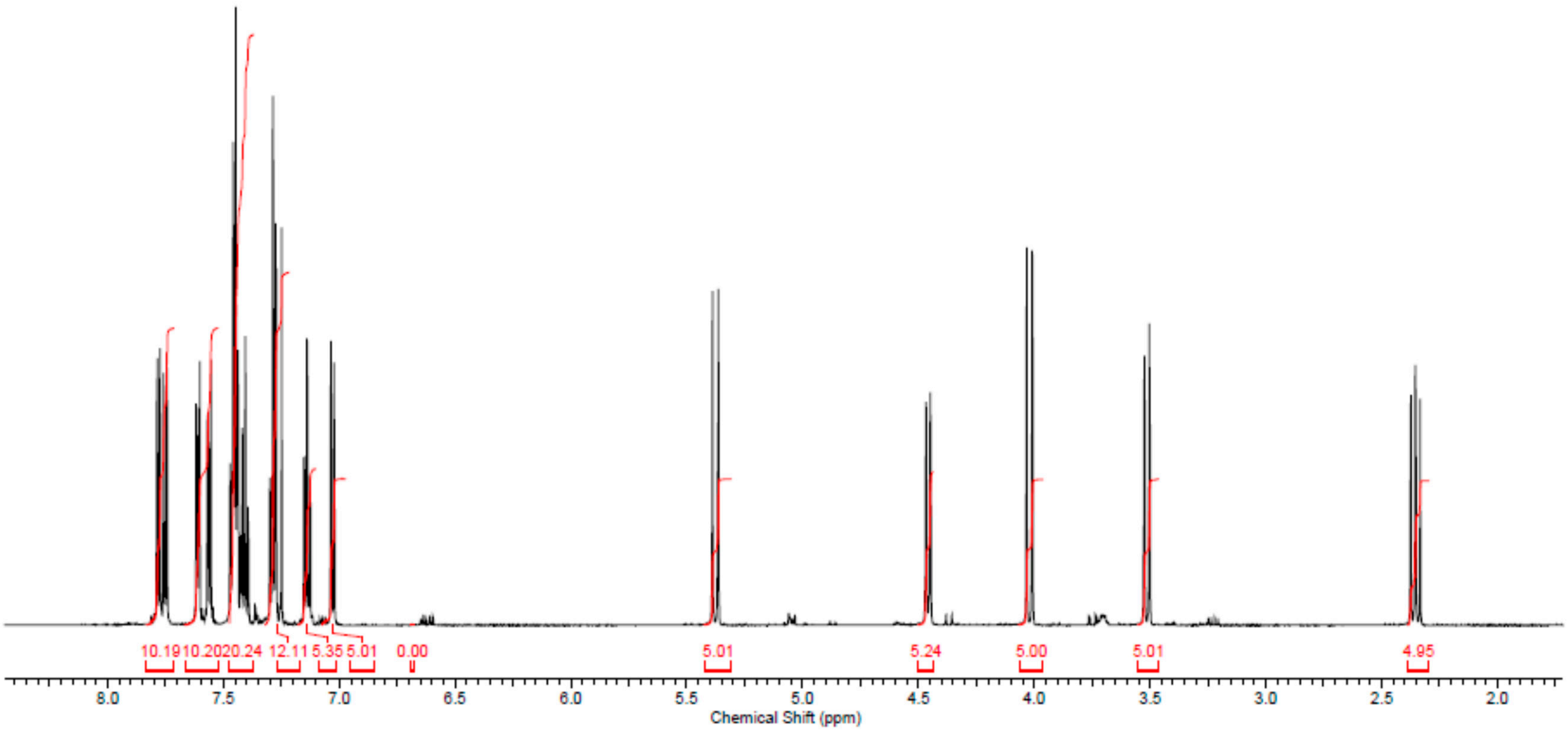

Figure S11. ^{1}H-NMR for compound **5e**.

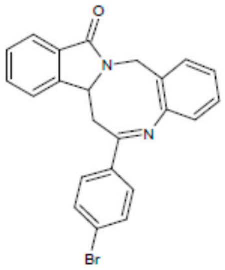

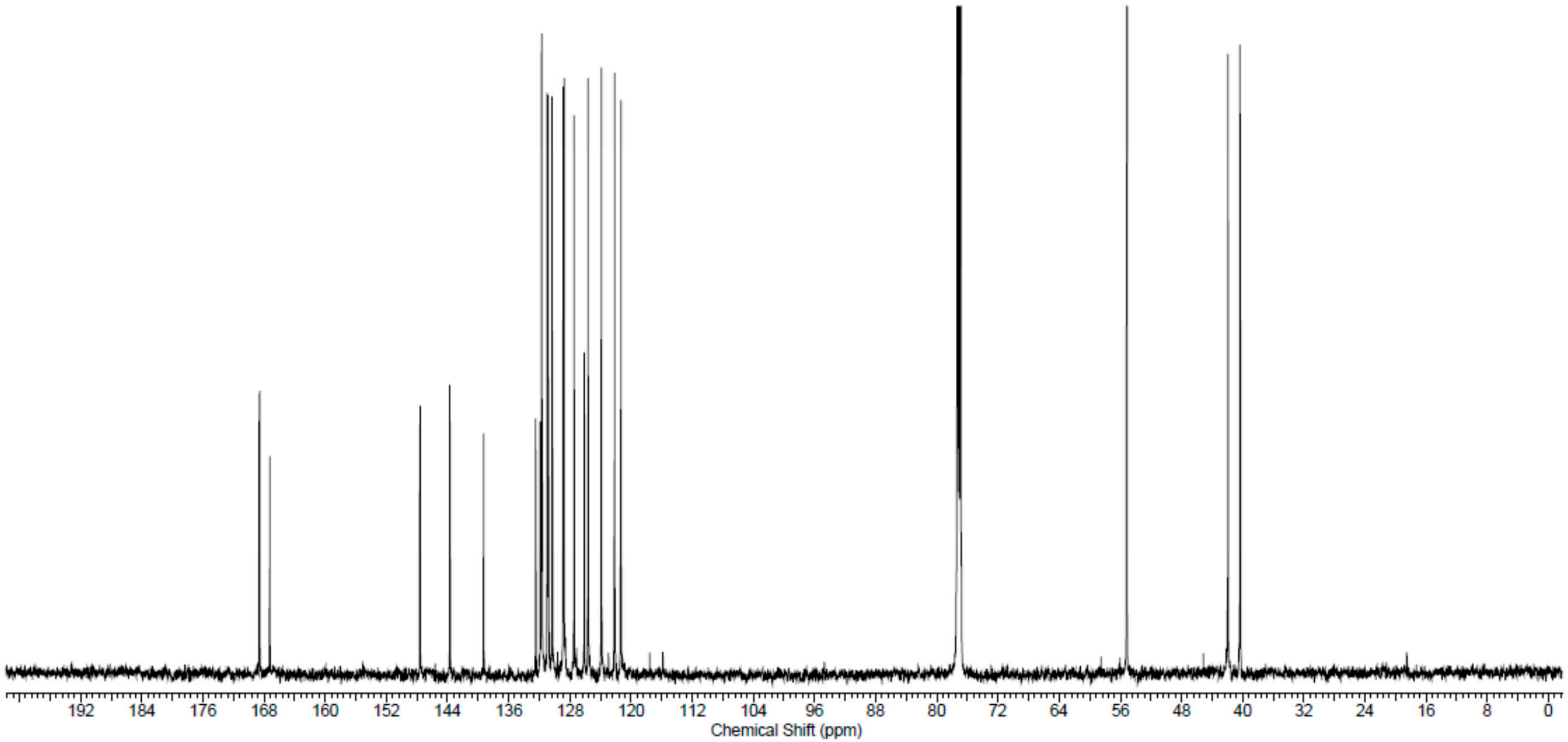

Figure S12. ^{13}C-NMRfor compound **5e**.

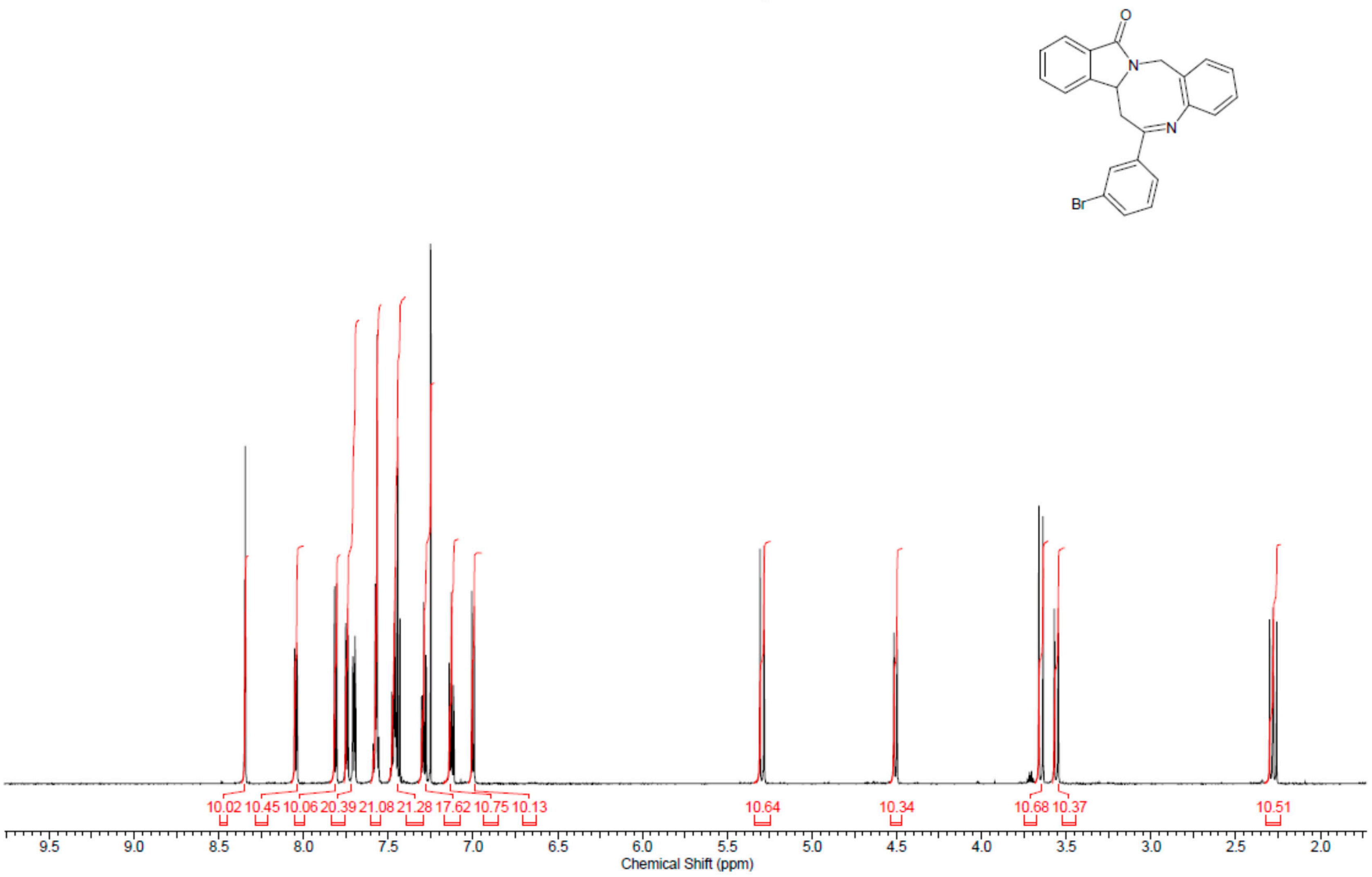

Figure S13. ^{1}H-NMR for compound **5f**.

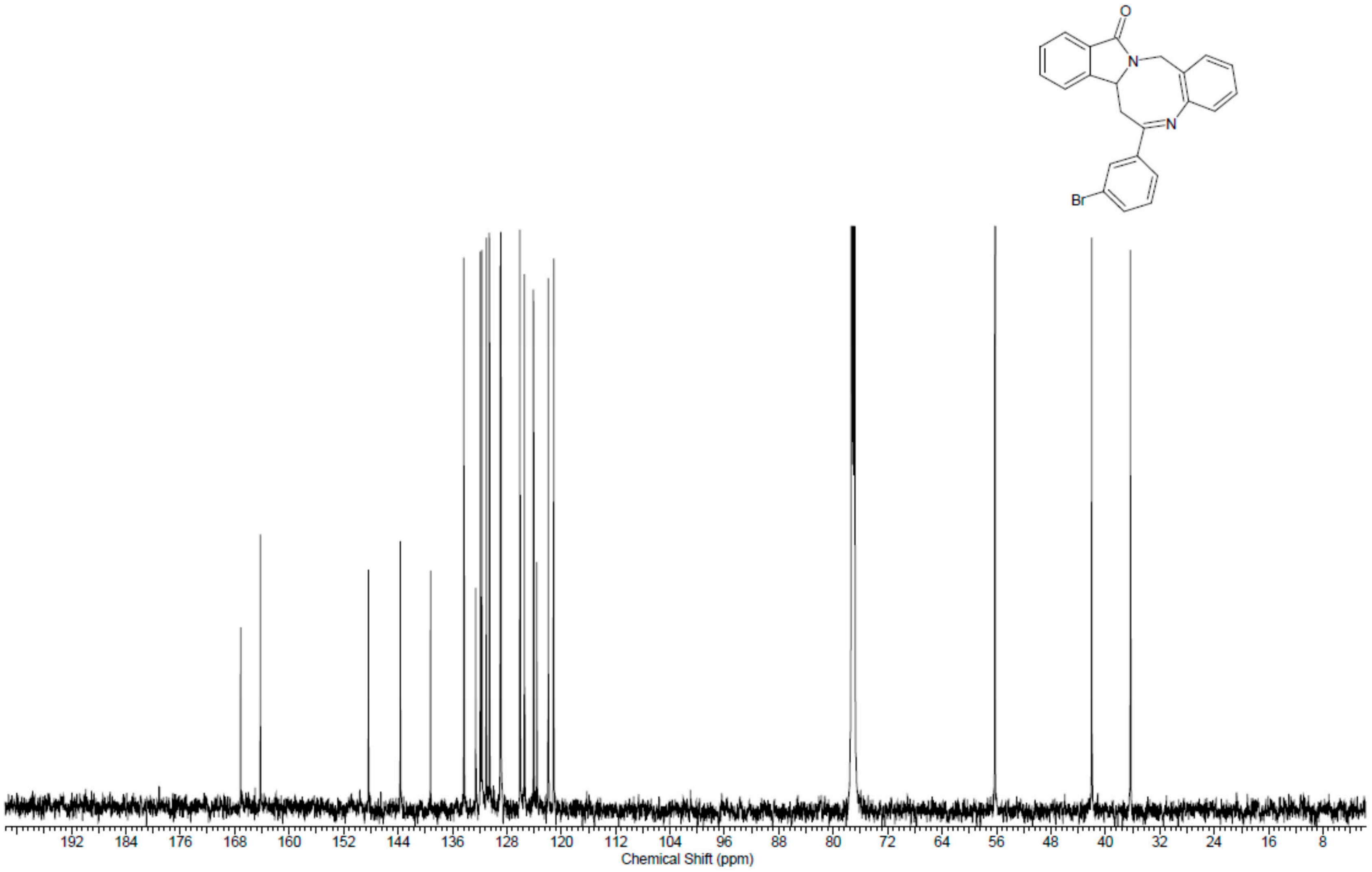

Figure S14. ^{13}C-NMRfor compound **5f**.

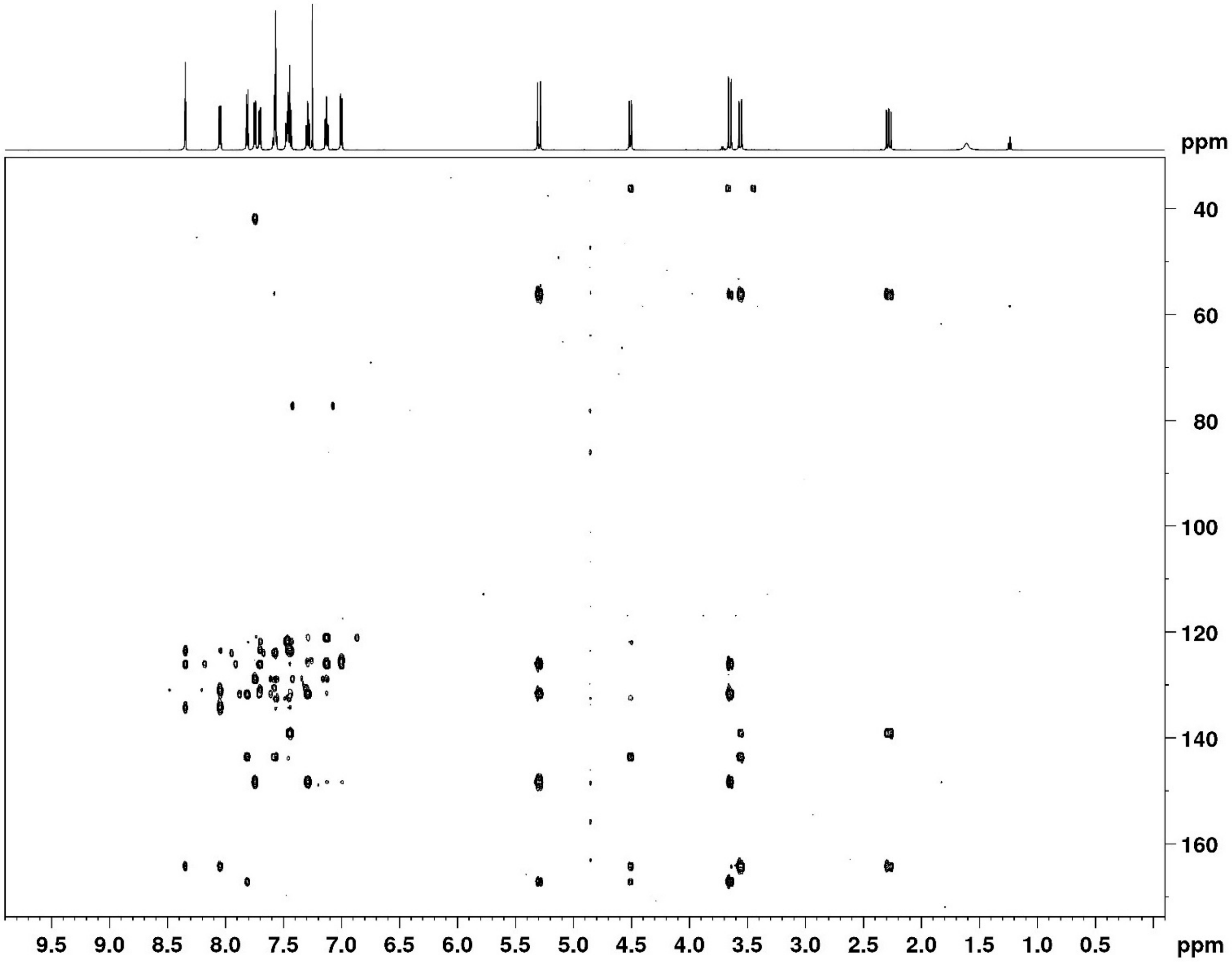

Figure S15. HMBC for compound **5f**.

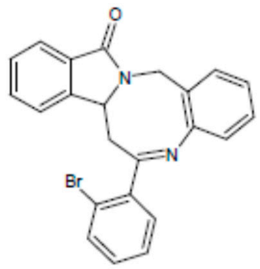

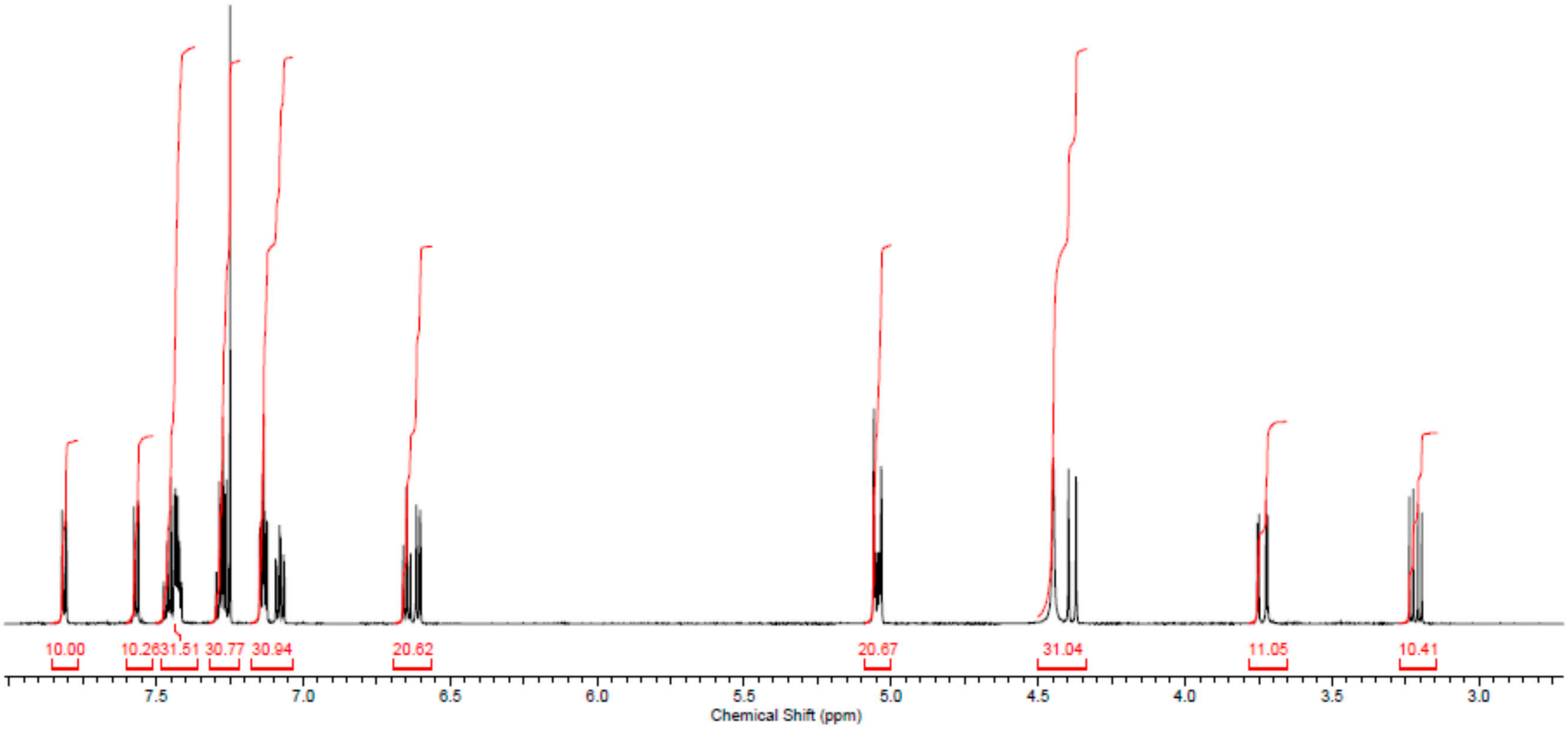

Figure S16. ^{1}H-NMR for compound **5g**.

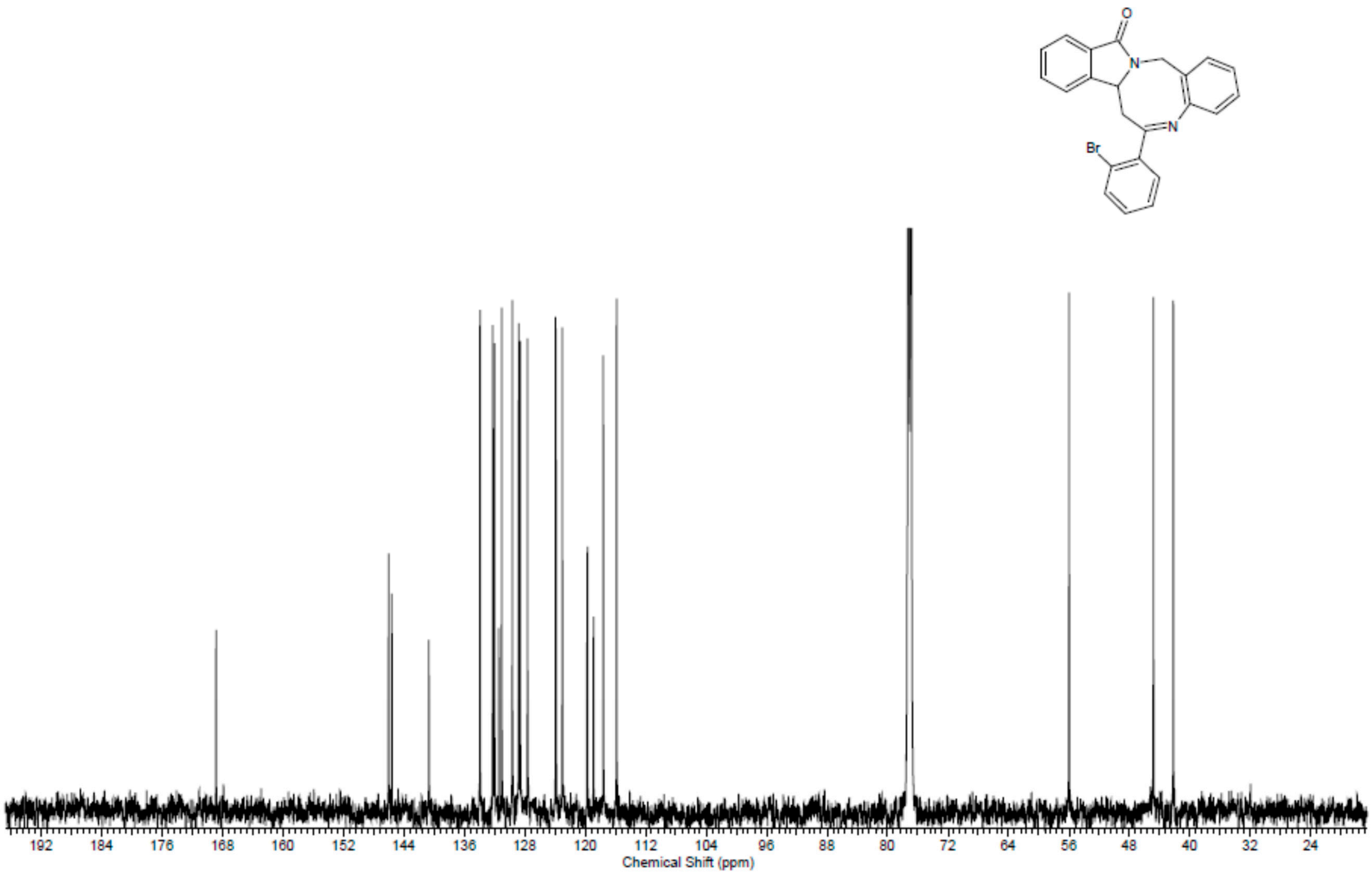

Figure S17. ^{13}C-NMRfor compound **5g**.

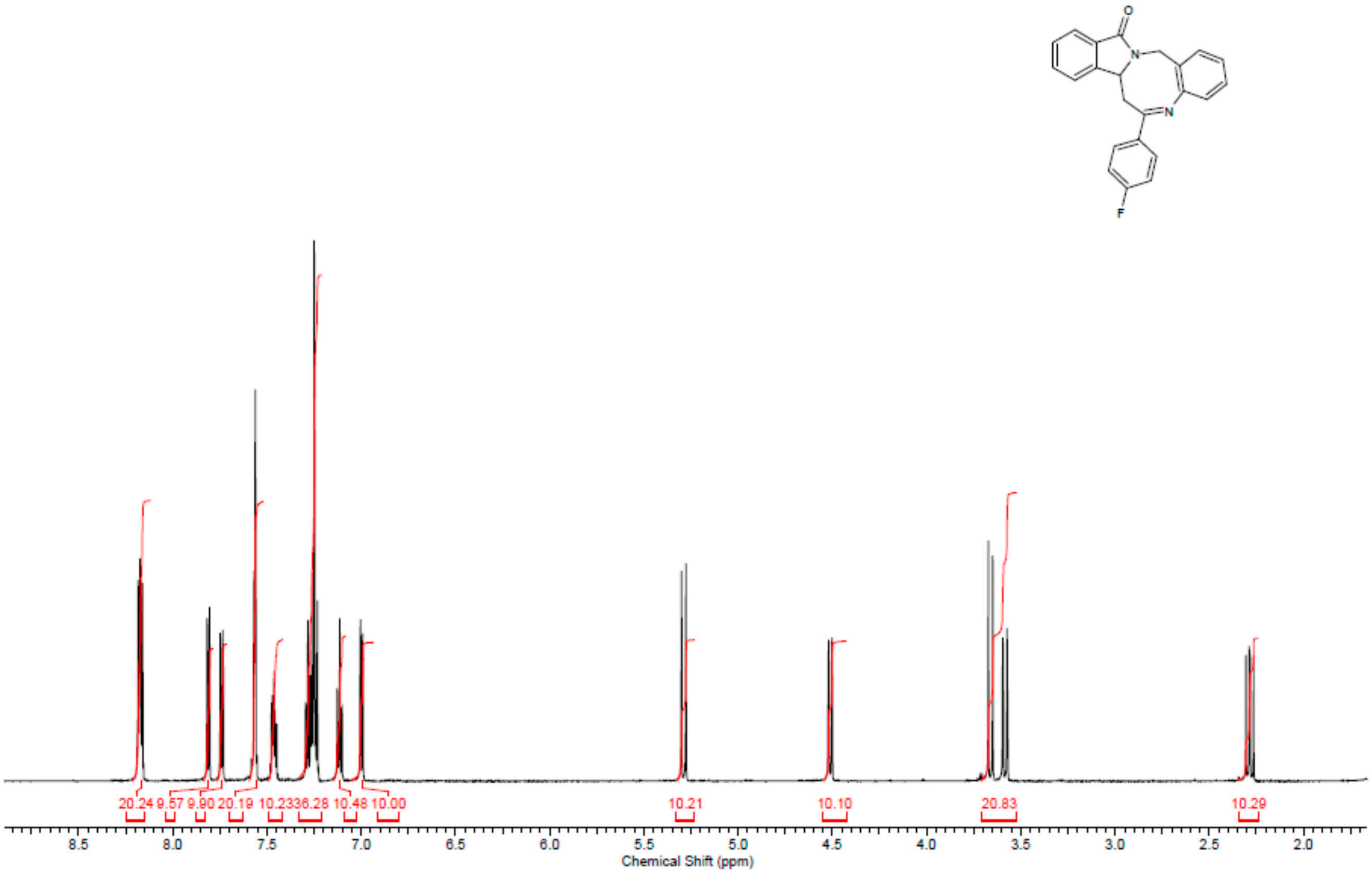

Figure S18. ¹H-NMR for compound **5h**.

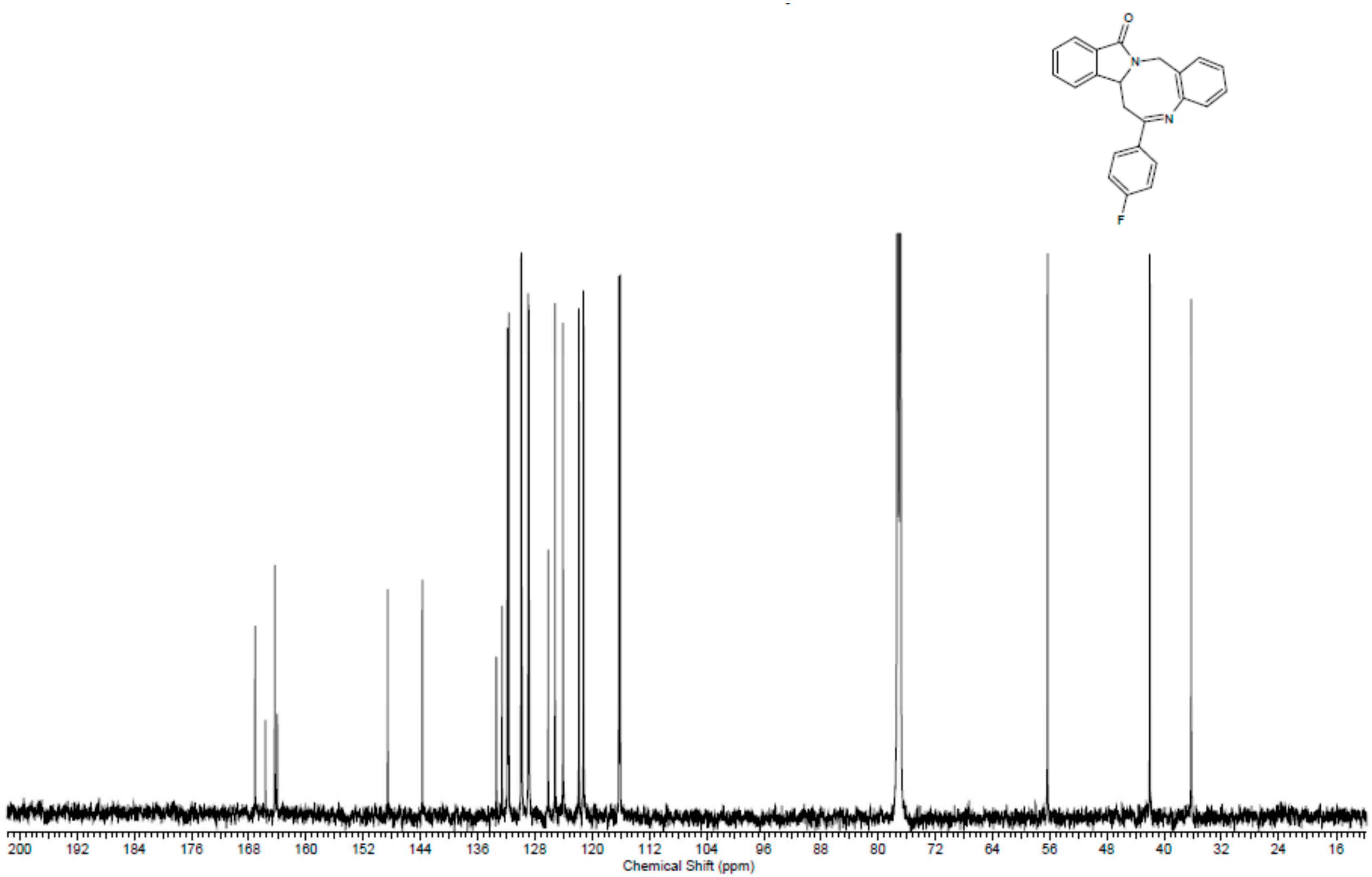

Figure S19. ^{13}C-NMRfor compound **5h**.

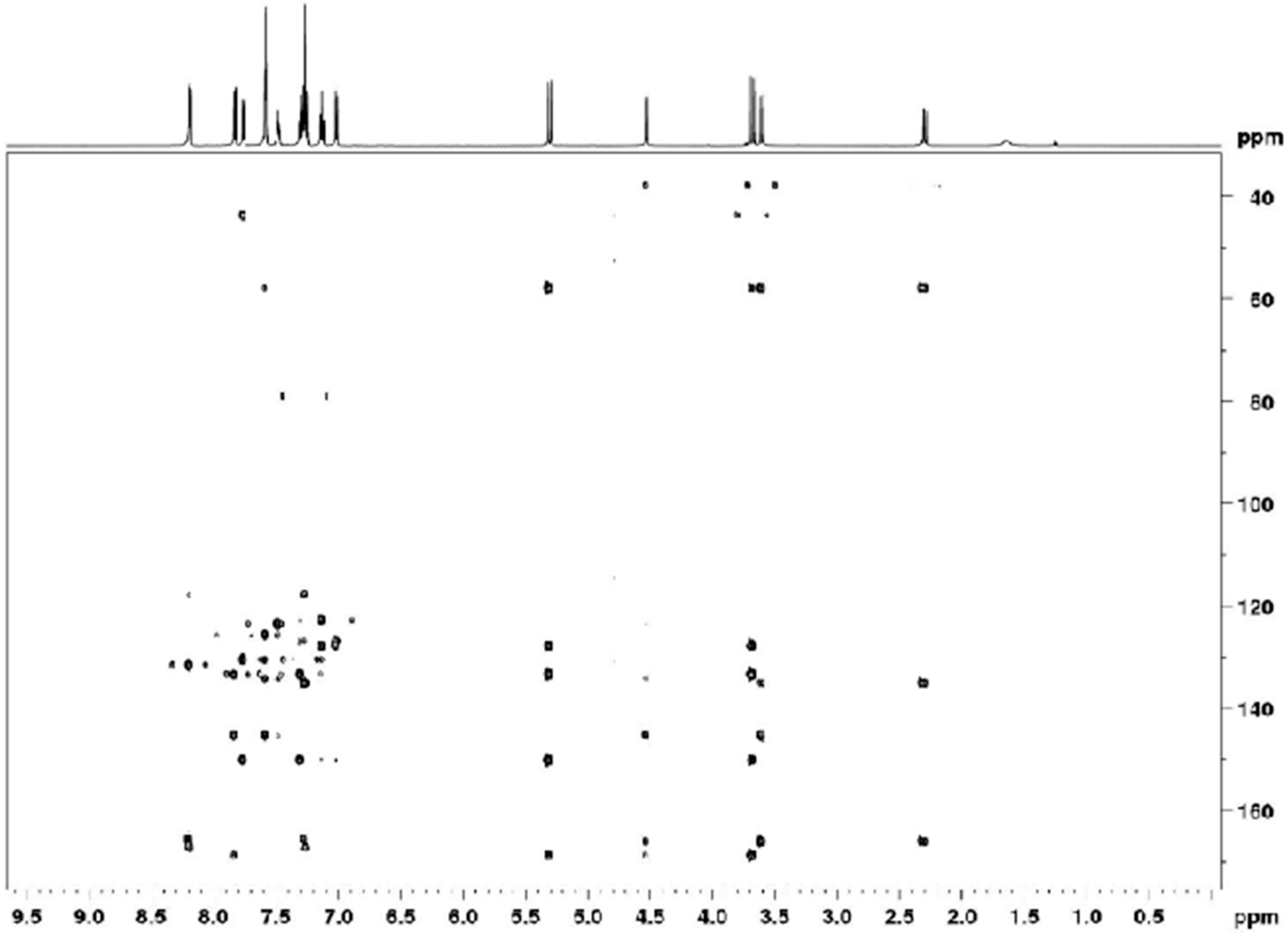

Figure S20. HMBC for compound **5h**.

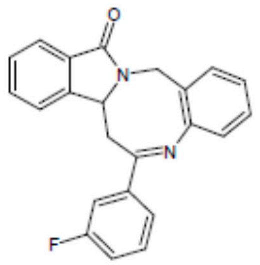

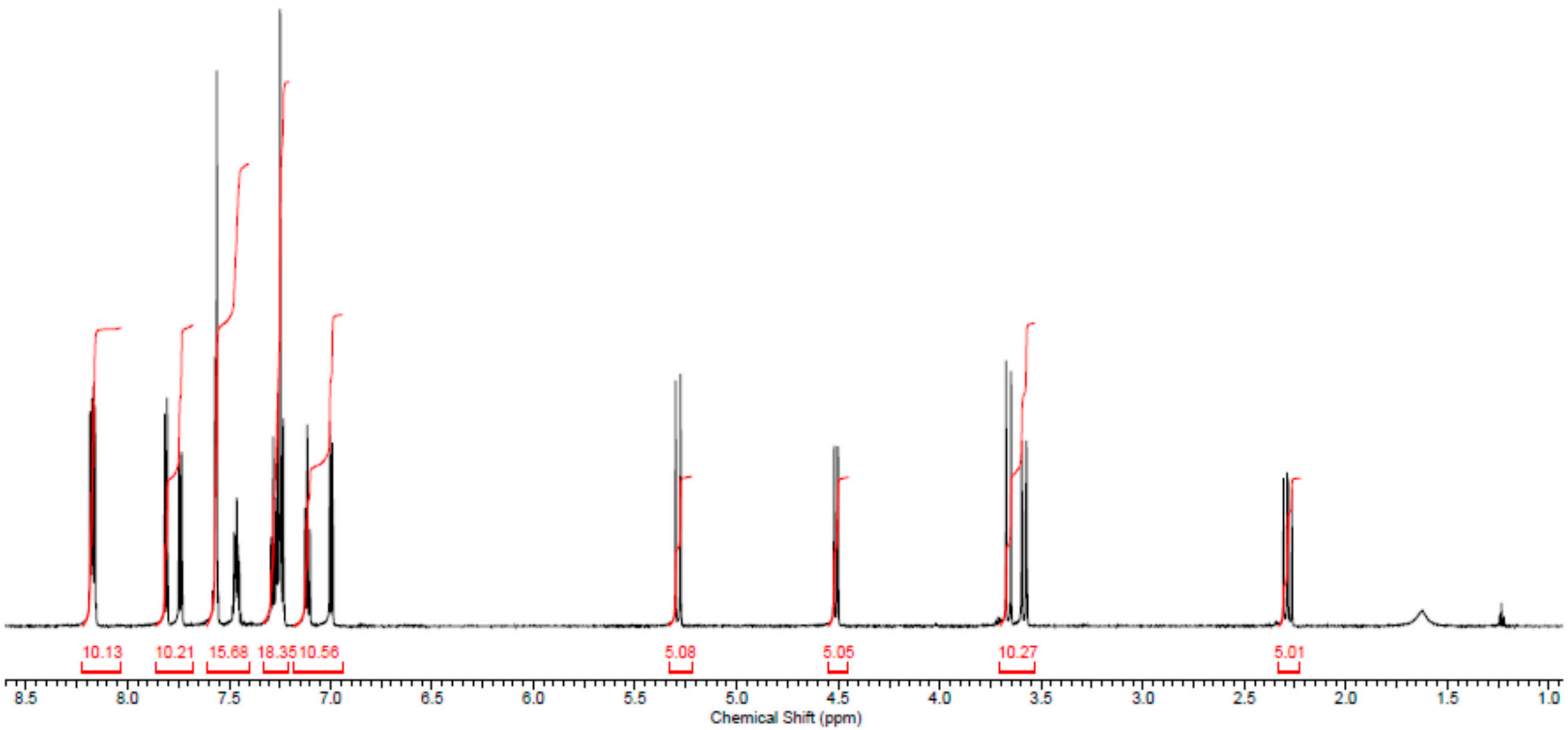

Figure S21. ^{1}H-NMR for compound **5i**.

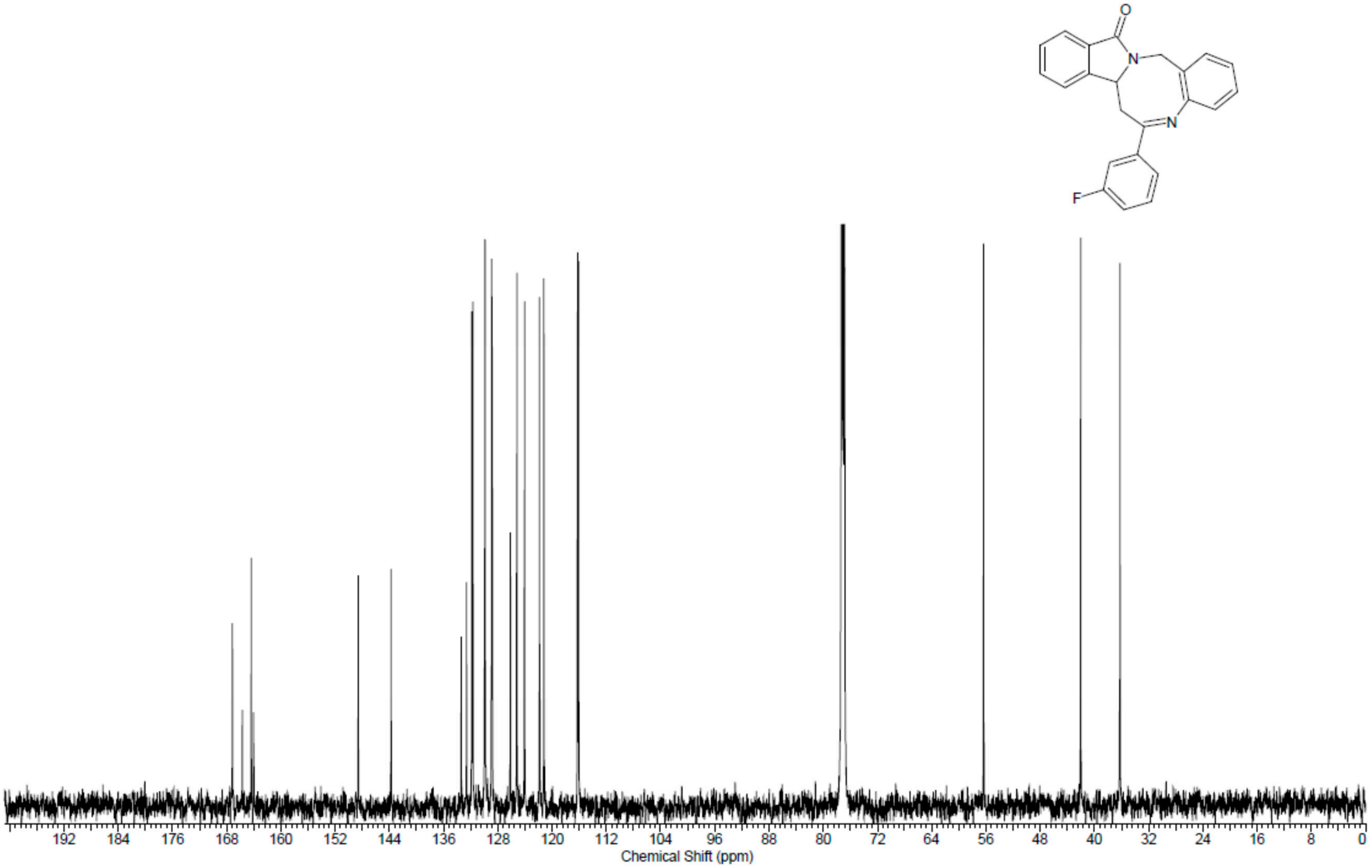

Figure S22. ^{13}C-NMRfor compound **5i**.

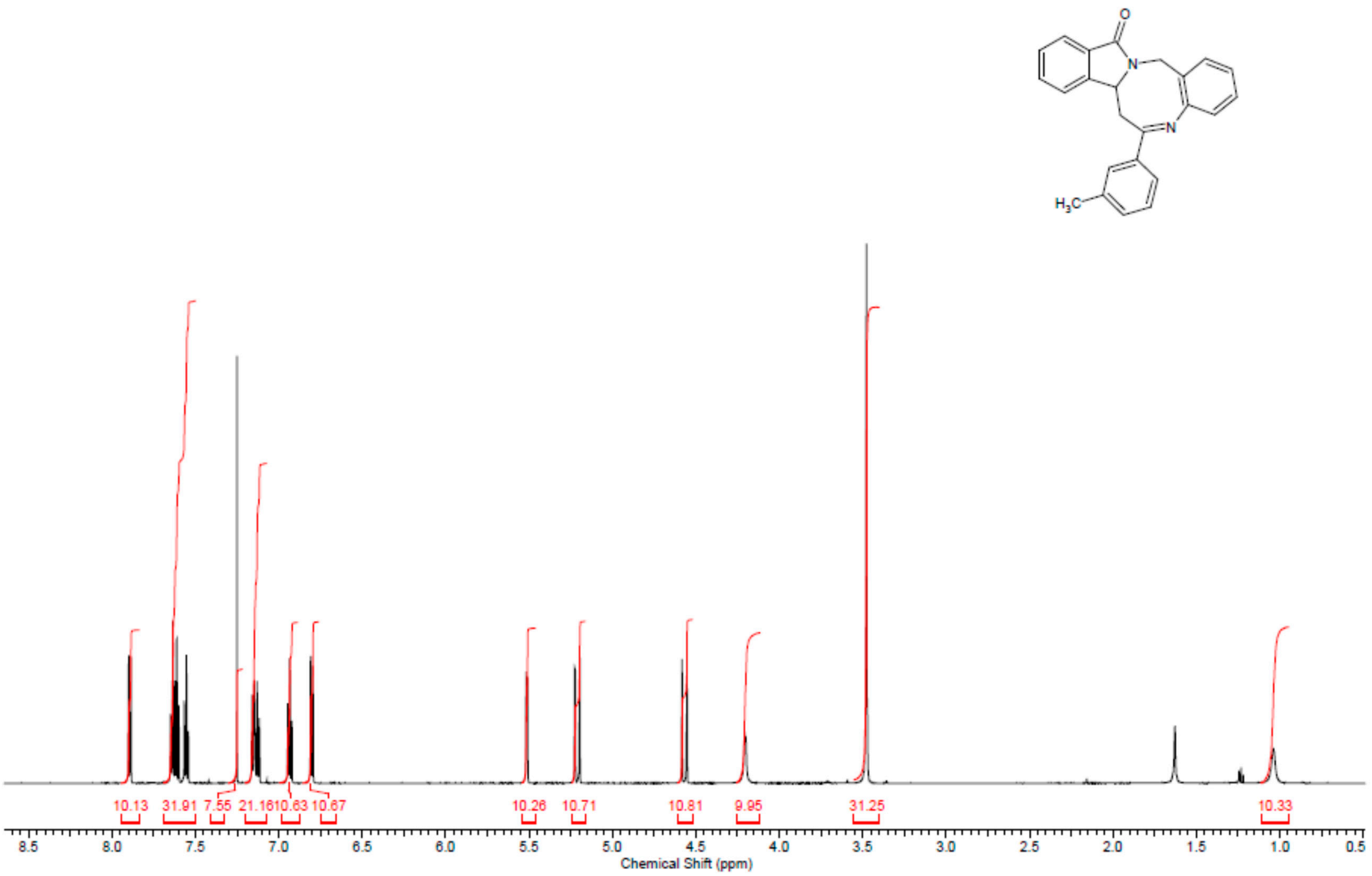

Figure S.23 ^{1}H-NMR for compound **5j**.

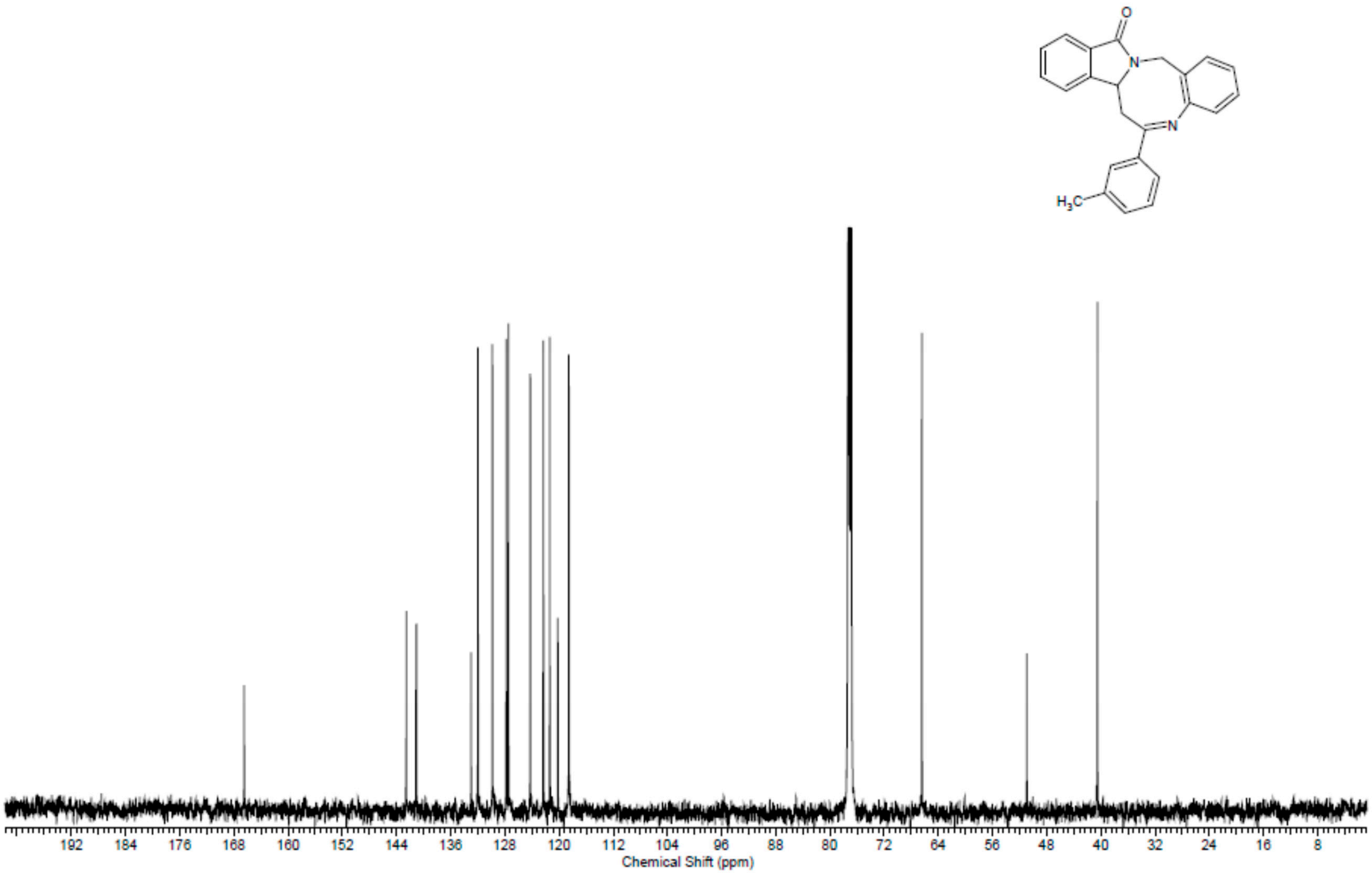

Figure S24. ^{13}C-NMRfor compound **5j**.

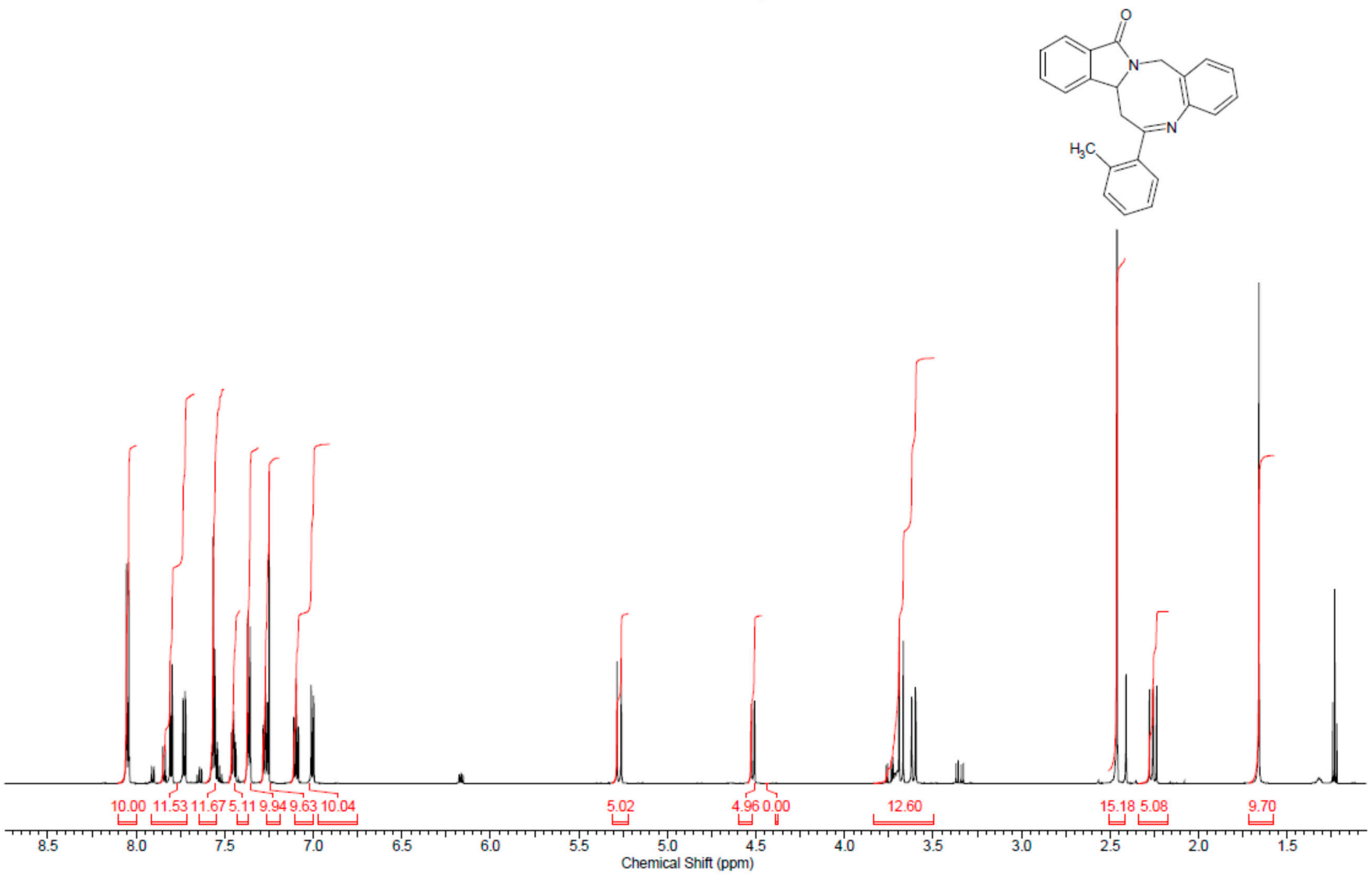

Figure S25. ^{1}H-NMR for compound **5k**.

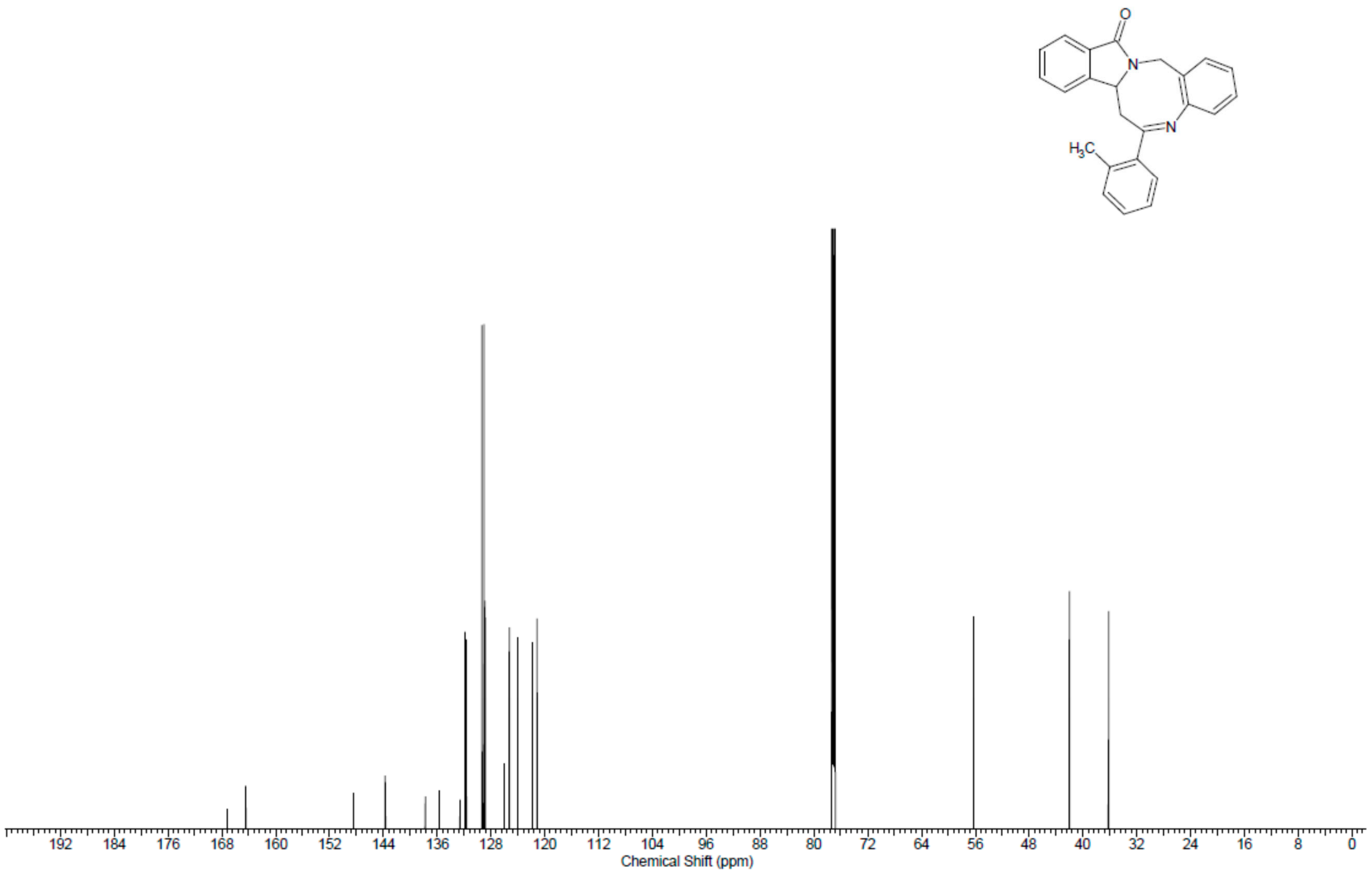

Figure S26. ^{13}C-NMRfor compound **5k**.

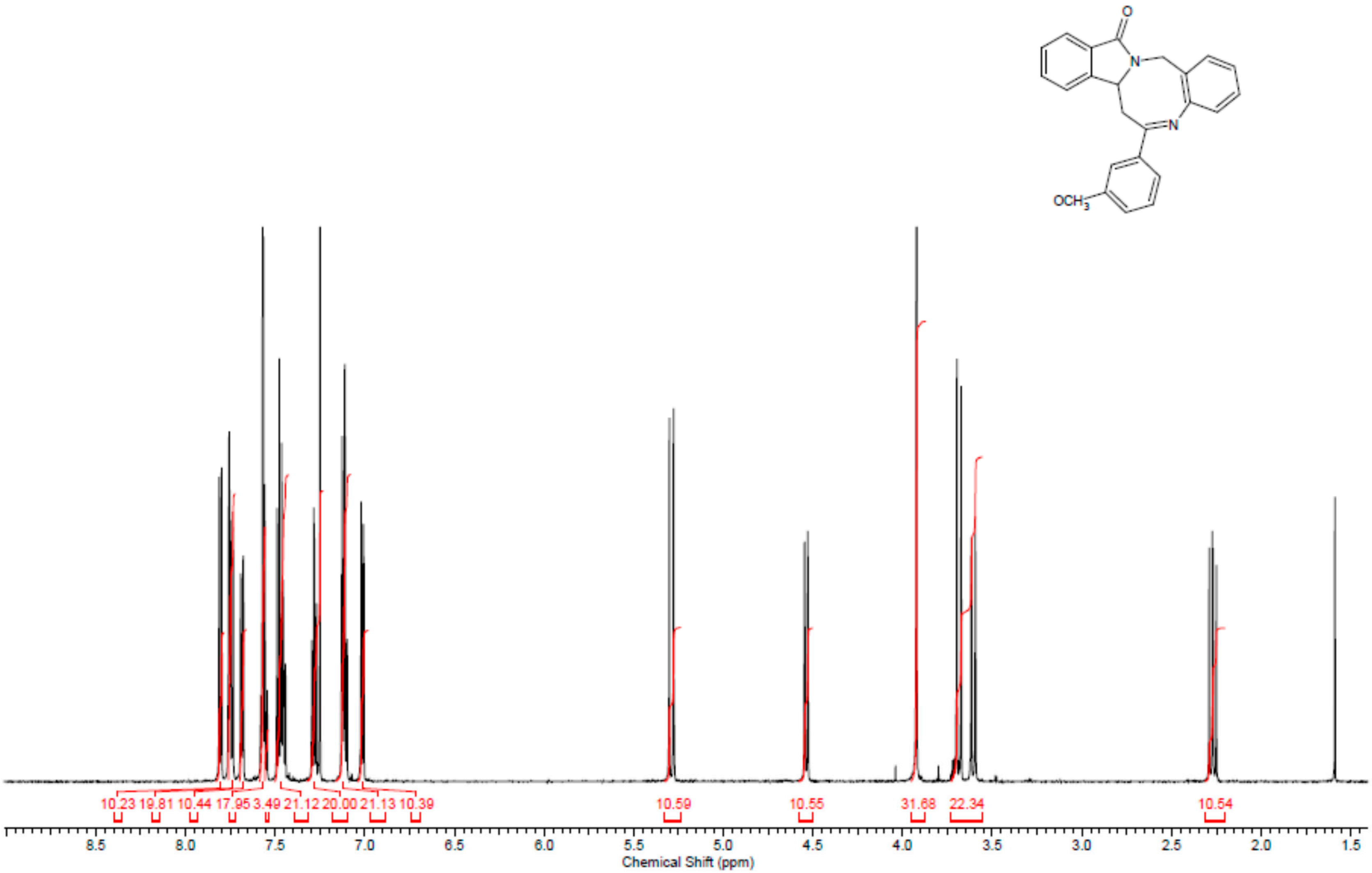

Figure S27. ^{1}H-NMR for compound **5l**.

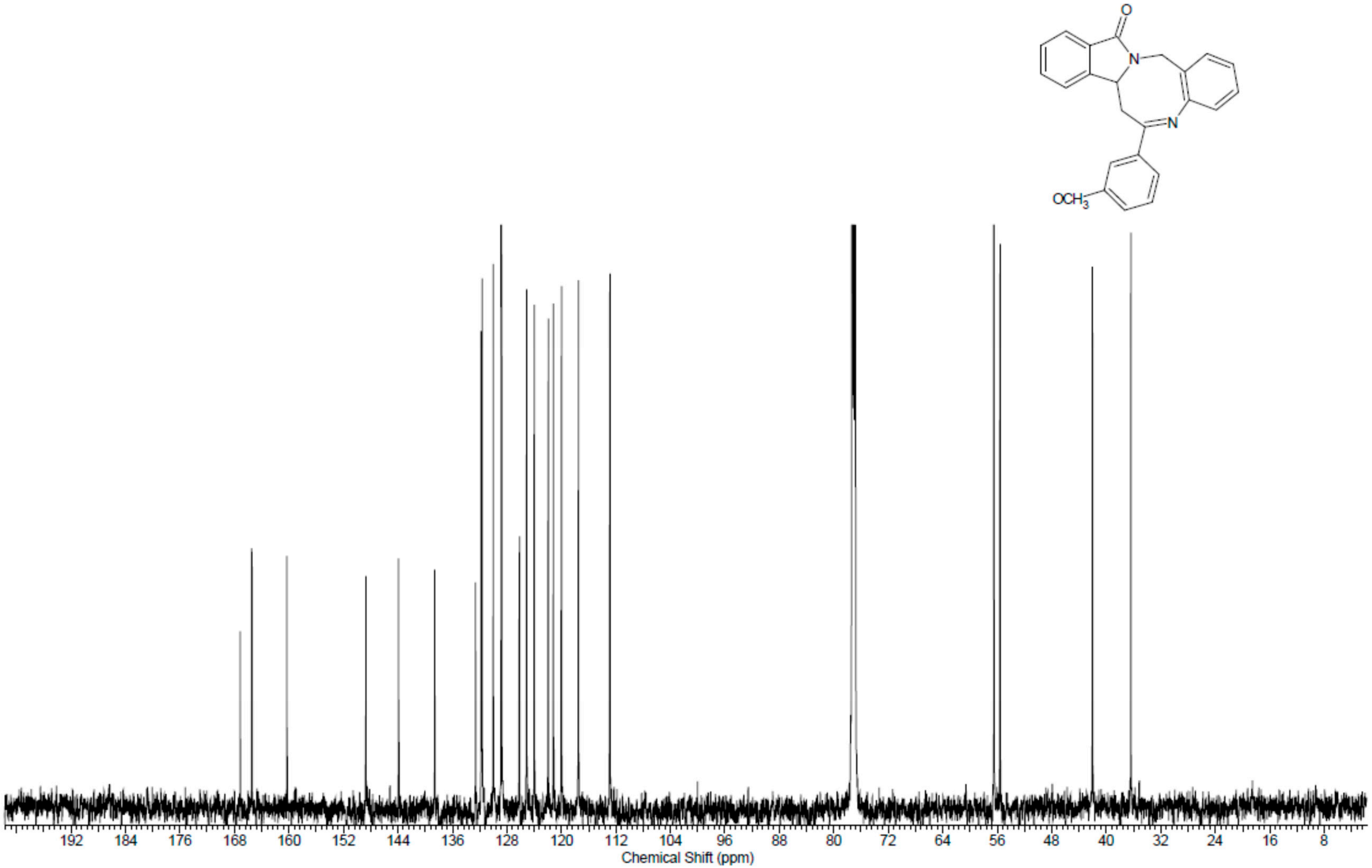

Figure S28. ^{13}C-NMRfor compound **5l**.

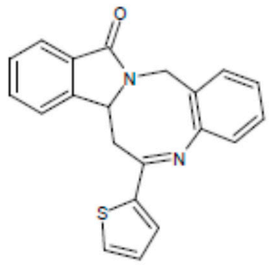

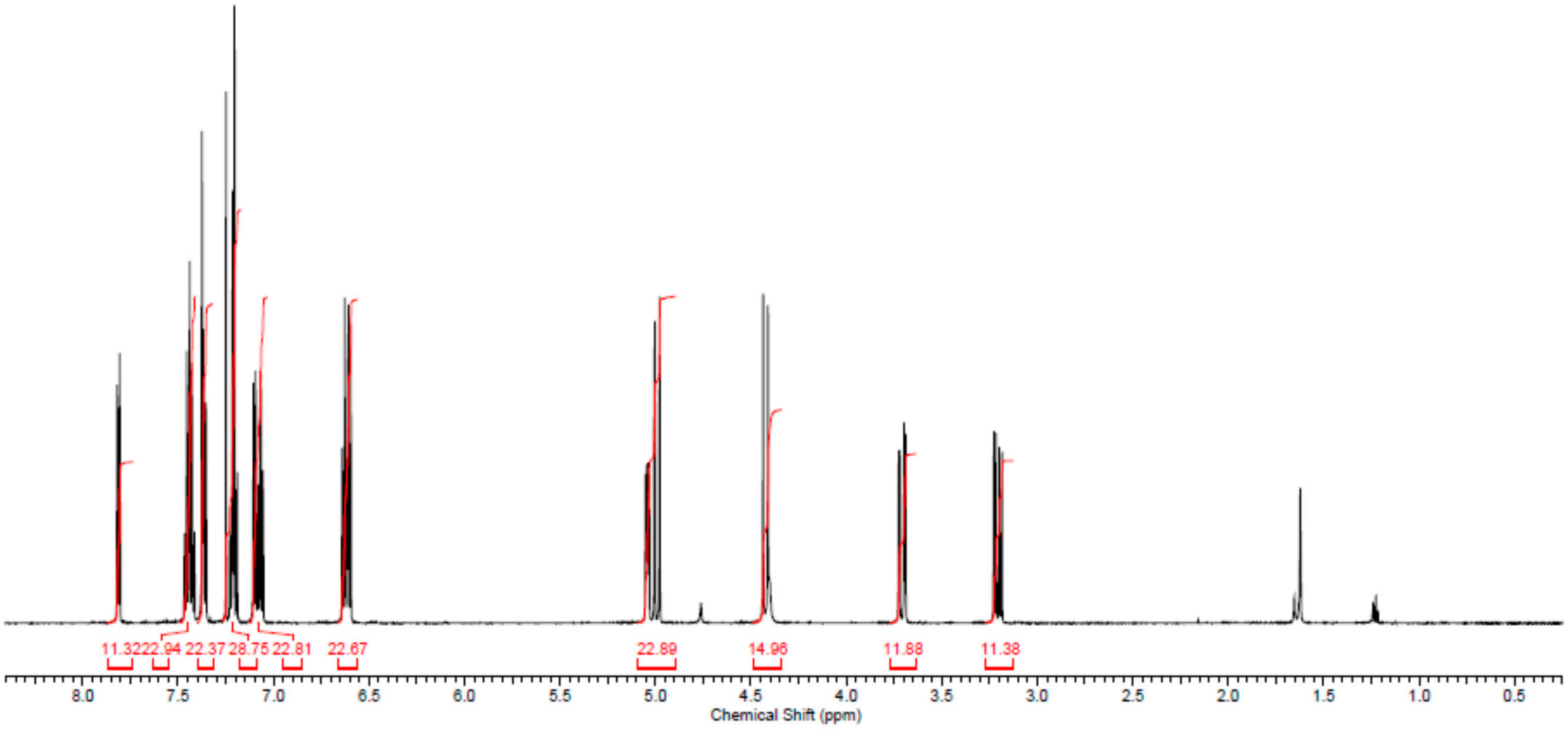

Figure S29. ^{1}H-NMR for compound **5m**.

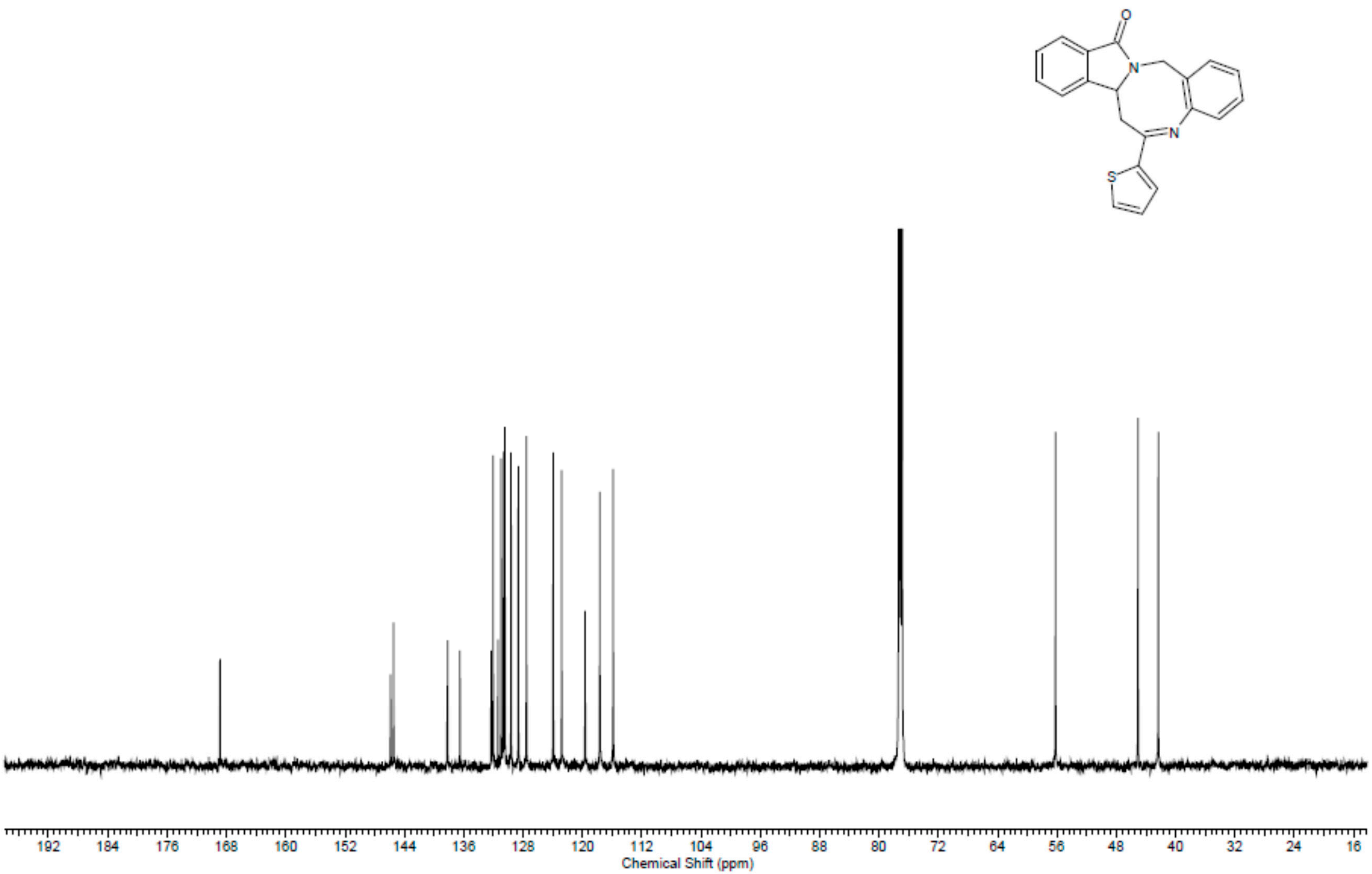

Figure S30. ^{13}C-NMRfor compound **5m**.
